# A Macroscopic Quantum Three-Box Paradox: Finding Consistency with Weak Macroscopic Realism

**DOI:** 10.3390/e25121620

**Published:** 2023-12-04

**Authors:** Channa Hatharasinghe, Manushan Thenabadu, Peter D. Drummond, Margaret D. Reid

**Affiliations:** Center for Quantum Science and Technology Theory, Swinburne University of Technology, Melbourne 3122, Australiapdrummond@swin.edu.au (P.D.D.)

**Keywords:** quantum paradox, macroscopic realism, non-invasive measurability, cat states

## Abstract

The quantum three-box paradox considers a ball prepared in a superposition of being in any one of three boxes. Bob makes measurements by opening either box 1 or box 2. After performing some unitary operations (shuffling), Alice can infer with certainty that the ball was detected by Bob, regardless of which box he opened, if she detects the ball after opening box 3. The paradox is that the ball would have been found with certainty by Bob in either box if that box had been opened. Resolutions of the paradox include that Bob’s measurement cannot be made non-invasively or else that realism cannot be assumed at the quantum level. Here, we strengthen the case for the former argument by constructing macroscopic versions of the paradox. Macroscopic realism implies that the ball is in one of the boxes prior to Bob or Alice opening any boxes. We demonstrate the consistency of the paradox with macroscopic realism, if carefully defined (as weak macroscopic realism, wMR) to apply to the system at the times prior to Alice or Bob opening any boxes but *after* the unitary operations associated with preparation or shuffling. By solving for the dynamics of the unitary operations and comparing with mixed states, we demonstrate agreement between the predictions of wMR and quantum mechanics: the paradox only manifests if Alice’s shuffling combines both local operations (on box 3) and nonlocal operations, on the other boxes. Following previous work, the macroscopic paradox is shown to correspond to a violation of a Leggett–Garg inequality, which implies failure of non-invasive measurability if wMR holds.

## 1. Introduction

The quantum three-box paradox [1] concerns the results inferred for a system at a time intermediate between two times where the system is in a pre-selected or a post-selected state [2]. The paradox was introduced by Aharonov and Vaidmann and has attracted much interest [3,4,5,6,7,8,9,10,11,12,13,14,15,16,17]. The paradox involves a ball prepared in a quantum superposition of being in any one of three boxes. Bob makes a measurement by opening either box 1 or box 2 to determine whether or not the ball is in the box he opens. Alice then makes specific transformations on the system by “shuffling” the ball among the boxes. After these operations, she knows that if she detects the ball in box 3 then Bob must have detected the ball in the box he opened. Paradoxically, the ball would have been found with certainty in either box if that box had been opened. The three-box paradox can be considered a quantum game, in which Alice can infer that Bob detected a particle with odds not possible classically [7,9].

The three-box paradox raised questions about what quantum effect is involved and how to resolve the paradox [3,4,5,6,7,8,9,11]. One response is to argue that for a system described as a superposition of quantum states; it cannot be assumed that the system is *in* one of those states until measured. Hence, it cannot be assumed that the ball is in one of the boxes prior to the box being opened. This approach defies the concept of *realism*. The argument is not so convincing for a macroscopic version of the paradox, where the states of the system are macroscopically distinct. *Macroscopic realism* (MR) would posit that the ball must be in one of the boxes irrespective of Alice or Bob opening a box. On the other hand, it could be argued that decoherence prevents the creation of macroscopic superposition states so that the macroscopic paradox cannot be realized. This motivates the challenge of a macroscopic version of the three-box paradox. The three-box paradox has been experimentally verified but at microscopic levels only [15,16,17].

In this paper, we present a mesoscopic three-box paradox corresponding to *N* quanta (the ball) in a superposition of being in one of three field modes (boxes). We also present a macroscopic paradox in which the states of the system correspond to macroscopically distinct coherent states of a single-mode field. Since macroscopic quantum superposition states have been created [18,19], we anticipate these predictions could be tested. This motivates consideration of other approaches in order to resolve the paradox consistently with macroscopic realism.

We show in this paper that the three-box paradox can be explained consistently with macroscopic realism *if* the definition of macroscopic realism is carefully refined as *weak macroscopic realism*. Following previous work [20,21,22,23], weak macroscopic realism (wMR) posits that the outcome of Bob’s or Alice’s measurement in opening any one of the boxes at time $t_{i}$ is predetermined: each box is either occupied by the ball or not, and the ball is in one of the boxes. The outcome of Alice or Bob opening box *K* is represented by a variable $\lambda_{K}$, which takes the value 1 or −1, if the ball is in the box *K* or not. Weak macroscopic realism posits specifically that prior to Alice or Bob opening the box *K*, this value is fixed at the time $t_{i}$ *once* any preparation and unitary transformations (shuffling) involving box *K* are completed. We refer to any such transformations as “local” to *K*. The definition of wMR also posits that the ball being in a particular box *K* at time $t_{i}$ is *not affected* by any measurement or shuffling that might occur solely for the other boxes *after* time $t_{i}$ [20,21,23]. Since the boxes may be spatially separated, we refer to such operations as “nonlocal”.

It is important to note that the definition of wMR is not concerned with microscopic details about the state of the ball. Hence, wMR does *not* posit that the ’state’ of the ball prior to Bob’s measurement is the same as the state after. In fact, for macroscopic superposition states, it has been argued that if the predetermination given by λ is valid, it is not possible to associate the ’state’ of the system given by a particular value of λ with any quantum state [20,24].

In this paper, we verify consistency with weak macroscopic realism for the results of the paradox, showing that (in a wMR-model) the paradox arises due to disturbance from Bob’s measurement, *but a measurable paradoxical effect occurs in the final joint probabilities only when* Alice’s further unitary transformations involve *both* the local box *K* and the other nonlocal boxes. We refer to this as a *local–nonlocal* operation. This allows us to test the predictions of weak macroscopic realism in a potential experiment. The dynamics of Alice’s transformations are modeled by specific interaction Hamiltonians and are illustrated by the *Q* function.

Our results support earlier conclusions that emphasize the role of Bob’s measurement disturbance in explaining the paradox [5,9,14]. Maroney pointed out that the conditions under which the paradox occurs are the same as those required to violate a Leggett–Garg inequality [9]. Leggett–Garg inequalities are derived from the assumptions of *macro-realism* [25]. Macro-realism posits macroscopic realism (MR) (that a system with two or more macroscopically distinct states available to it must be *in* one of those states) and macroscopic noninvasive measurability (NIM)—that it is possible to measure which of the two macroscopically distinct states the system is in with negligible disturbance to the future dynamics of the system. Violation of the inequalities can therefore arise from a failure of NIM and be consistent with MR. By illustrating the violation of a new class of Leggett–Garg inequalities, Maroney argues that the quantum feature of the three-box paradox is measurement disturbance that cannot be explained classically. In this paper, we show that the negation of macro-realism is possible for the macroscopic and mesoscopic versions of the three-box paradox, along the lines proposed by Maroney, thereby illustrating the quantum nature of the proposed experiments. Similarly, Blasiak and Borsuk identify causal structures for the three-box paradox, showing that a realist viewpoint necessitates measurement disturbance in order to maintain consistency with the assumption of realism [14].

Our model extends previous work since a parameter α or *N* is introduced, which quantifies the level of ’macroscopic distinctness’ of the states of the system, corresponding to the ball being in a box or not. The disturbance to the system due to Bob’s measurement can be evaluated by comparing the quantum states before and after. We find that the *Q* functions for the two states become identical as the system becomes macroscopic (α→∞). This supports Leggett and Garg’s macro-realism premise that a noninvasive measurement should exist for a sufficiently large system. However, when we evaluate the predictions for Alice detecting a ball in box 3, we find that the difference between the predictions, depending on whether Bob makes a measurement or not, remains macroscopically evident even as α→∞. The paradoxical results arise from microscopic differences existing at a prior time, similar to a quantum revival.

The results of this paper also support the work of Thenabadu et al. [26] and Thenabadu and Reid [20,27], who gave predictions for violations of Leggett–Garg and Bell inequalities involving the macroscopically distinct coherent states |α〉 and |−α〉. These authors showed how the violations can be consistent with wMR, demonstrating similarly that the Bell-nonlocal effect arises when the unitary transformations that determine the measurement settings are carried out at *both* locations (in a *local–nonlocal* operation) [21]. Similar results are obtained in [21,23,28].

The layout of the paper is as follows. In Section 2, we summarize the three-box paradox. In Section 3, we present a mesoscopic paradox where the three boxes are distinct modes, and the ball corresponds to *N* quanta. A macroscopic (modified) version of the paradox involving macroscopically distinct coherent states (cat states) is presented in Section 4. In Section 5, we give the definition of wMR and show consistency with wMR for both versions of the paradox. The violation of the Leggett–Garg inequalities is demonstrated for the mesoscopic and macroscopic three-box paradox, in Section 6.

## 2. Three-Box Paradox

We first summarize the states, transformations, and measurements involved in the paradox [1]. The system has three boxes and one ball. The state of the ball in box *K* is denoted |k〉. The system is prepared at time $t_{0}$ with the ball in box three, i.e., in state |3〉. A unitary transformation $U_{i}$ transforms the initial system into the superposition state

(1) $$|\psi_{sup}\rangle \equiv |\psi_{1}\rangle =\frac{1}{\sqrt{3}}(|1\rangle +|2\rangle +\left|3\rangle \right)$$

at time $t_{1}$, so that $U_{i}\left|3\rangle =\right|\psi_{sup}\rangle$. Using the basis set {|1〉,|2〉, |3〉}, we find

(2) $$\begin{array}{ccc} U_{i} & = & \frac{1}{\sqrt{6}}\left(\begin{array}{ccc} \sqrt{3} & 1 & \sqrt{2}\\ \sqrt{3} & 1 & \sqrt{2}\\ 0 & 2 & \sqrt{2} \end{array}\right) \end{array}$$

where the basis states |k〉 correspond to column matrices $\left(a_{j1}\right)$ with coefficients given as $a_{j1}=\delta_{jk}$. The form of $U_{i}$ is justified by noting that the transformation can be physically carried out by first creating from |3〉 the superposition $\sqrt{\frac{2}{3}}|2\rangle +\frac{1}{\sqrt{3}}|3\rangle$. This is achieved by the unitary operation

(3) $$U_{1i}=\frac{1}{\sqrt{3}}\left(\begin{array}{ccc} \sqrt{3} & 0 & 0\\ 0 & 1 & \sqrt{2}\\ 0 & \sqrt{2} & 1 \end{array}\right).$$

which leaves the component for state |1〉 unchanged. A second transformation

(4) $$U_{2i}=\frac{1}{\sqrt{2}}\left(\begin{array}{ccc} -1 & 1 & 0\\ 1 & 1 & 0\\ 0 & 0 & \sqrt{2} \end{array}\right)$$

then creates the superposition $\frac{1}{\sqrt{2}}(|2\rangle +\left|1\rangle \right)$ from |2〉, leaving the third-state component unchanged. Hence, $U_{i}=U_{2i}U_{1i}$.

Now, Bob can make a measurement to determine whether the system is in |1〉 (the ball in box 1) or not. Assuming Bob makes an ideal projective measurement, the state of the system after the measurement according to quantum mechanics is |1〉 if he detects the ball in box 1. Otherwise, the system is in the superposition state $\frac{1}{\sqrt{2}}(|2\rangle +\left|3\rangle \right)$. Alternatively, Bob may make a measurement to determine if the system is in state |2〉 (the ball is in box 2) or not. Assuming an ideal projective measurement, the state of the system after the measurement according to quantum mechanics is |2〉 if he detects the ball in box 2. Otherwise, the system is in the superposition state $\frac{1}{\sqrt{2}}(|1\rangle +\left|3\rangle \right)$.

After the interactions given by Bob’s measurements, at time $t_{2}$ Alice makes further measurements, to post-select for the state

(5) $$|\psi_{f}\rangle =\frac{1}{\sqrt{3}}(|1\rangle +|2\rangle -\left|3\rangle \right)$$

which is orthogonal to both $\frac{1}{\sqrt{2}}(|2\rangle +\left|3\rangle \right)$ and $\frac{1}{\sqrt{2}}(|1\rangle +\left|3\rangle \right)$. The measurement is realized as a transformation $U_{f}$, so that $|\psi_{f}\rangle$ maps to |3〉 at time $t_{3}$. Alice then performs the post-selection by determining whether the ball is in box 3 at time $t_{3}$. $U_{f}$ has the property that its inverse satisfies

(6) $$U_{f}^{-1}\left|3\rangle =\right|\psi_{f}\rangle .$$

To find $U_{f}$, we follow the above procedure and first apply the unitary transformation $U_{1f}$ that transforms |3〉 into the superposition $\sqrt{\frac{2}{3}}|2\rangle -\frac{1}{\sqrt{3}}|3\rangle$. Then, we create superposition $\frac{1}{\sqrt{2}}(|2\rangle +\left|1\rangle \right)$ from |2〉, which defines $U_{2f}=U_{2i}$. We find

(7) $$U_{1f}=\frac{1}{\sqrt{3}}\left(\begin{array}{ccc} \sqrt{3} & 0 & 0\\ 0 & 1 & \sqrt{2}\\ 0 & \sqrt{2} & -1 \end{array}\right).$$

Hence, $U_{f}^{-1}=U_{2f}U_{1f}$. Hence, the required transformation is $U_{f}=U_{1f}^{-1}U_{2f}^{-1}$, which, noting that the transformations are unitary so that $U_{2f}^{-1}=U_{2f}$ and $U_{1f}^{-1}=U_{1f}$, becomes $U_{f}^{-1}\left|3\rangle =\right|\psi_{f}\rangle$. Hence, Alice’s transformation is

(8) $$\begin{array}{ccc} U_{f} & = & \frac{1}{\sqrt{6}}\left(\begin{array}{ccc} -\sqrt{3} & \sqrt{3} & 0\\ 1 & 1 & 2\\ \sqrt{2} & \sqrt{2} & -\sqrt{2} \end{array}\right). \end{array}$$

If after his measurements Bob determines the system to be in |1〉, then the output after Alice’s transformations is

(9) $$\begin{array}{ccc} U_{f}|1\rangle & = & \frac{1}{\sqrt{6}}\left(\begin{array}{c} -\sqrt{3}\\ 1\\ \sqrt{2} \end{array}\right). \end{array}$$

If Bob measures that the system is not in |1〉, then the output after Alice’s operations is

(10) $$\begin{array}{ccc} U_{f}\frac{1}{\sqrt{2}}\left(\begin{array}{c} 0\\ 1\\ 1 \end{array}\right) & = & \frac{1}{2\sqrt{3}}\left(\begin{array}{c} \sqrt{3}\\ 3\\ 0 \end{array}\right). \end{array}$$

If after his measurements Bob determined the system to be in |2〉, then the final state is

(11) $$\begin{array}{ccc} U_{f}|2\rangle & = & \frac{1}{\sqrt{6}}\left(\begin{array}{c} \sqrt{3}\\ 1\\ \sqrt{2} \end{array}\right). \end{array}$$

If Bob measures that the system is not in |2〉, then the final state is

(12) $$\begin{array}{ccc} U_{f}\frac{1}{\sqrt{2}}\left(\begin{array}{c} 1\\ 0\\ 1 \end{array}\right) & = & \frac{1}{2\sqrt{3}}\left(\begin{array}{c} -\sqrt{3}\\ 3\\ 0 \end{array}\right). \end{array}$$

We see that if Bob determines that the system is not in state |1〉 (or |2〉), then the probability of Alice determining that the system is in state |3〉 at time $t_{3}$ is zero. This leads to the paradox.

The measured probabilities for the paradox can be summarized. We follow the notation used in previous papers [9], where $I_{k}$ represents that the ball is found in box I at time $t_{k}$, and $-I_{k}$ represents that the ball was not found in box I at time $t_{k}$. The subscripts BI denote that box *I* was opened by Bob. The subscript *A* denotes that Alice opens box 3 at time $t_{3}$. The probabilities for the detection of a ball if Bob opens the box 1 is $P_{B1}\left(1_{2}\right)=1/3$. Similarly, $P_{B2}\left(2_{2}\right)=1/3$. Here, we have denoted for convenience $t_{2}$ as the time of Bob’s opening the box, whereas in fact in our description, Bob performed the measurement at a time *t* just after the preparation of the state $|\psi_{sup}\rangle$ at $t_{1}$, and before $t_{2}$, when Bob’s measurement was complete. The probabilities are equivalently denoted by either subscript since $P_{BI}\left(1_{2}\right)\equiv P_{B1}\left(1_{1}\right)$ and $P_{B2}\left(2_{2}\right)=P_{B2}\left(2_{1}\right)$, etc. From (9), if Bob detects the ball in box 1, then $P_{B1,A}\left(3_{3}|1_{2}\right)=1/3$. If Bob opens box 1, the joint probabilities are $P_{B1,A}\left(1_{2},3_{3}\right)=P_{B1,A}\left(3_{3}|1_{2}\right)P_{B1}\left(1_{2}\right)=1/9$. Also, if Bob opens box 1, then $P_{B1}\left(3_{3}\right)=1/9$. Hence, $P_{B1,A}\left(1_{2}|3_{3}\right)=1$. Similarly, if Bob opens box 2, the probability of him detecting the ball given Alice detects a ball in box 3 is $P_{B2,A}\left(2_{2}|3_{3}\right)=1$. We find

(13) $$P_{B1,A}\left(1_{2}|3_{3}\right)=P_{B2,A}\left(2_{2}|3_{3}\right)=1.$$

Whenever, Alice measures that the ball is in box 3 (i.e., when she confirms the system at time $t_{3}$ is in the state |3〉), it is certain that Bob found the ball in the box he measured. This gives the paradox since, if Bob’s measurement is non-disturbing, it would be concluded that the ball was in both boxes with certainty at time $t_{1}$, just before his measurement.

The aspect of measurement disturbance can be studied. If there is no measurement by Bob, then the final state at time $t_{3}$ after Alice’s operations is

(14) $$\begin{array}{ccc} U_{f}|\psi_{sup}\rangle & = & \frac{1}{3\sqrt{2}}\left(\begin{array}{c} 0\\ 4\\ \sqrt{2} \end{array}\right). \end{array}$$

The probability of Alice detecting a ball if Bob makes no measurement is $P_{N}\left(3_{3}\right)=1/9$. We note that

(15) $$P_{N,A}\left(3_{3}\right)=P_{B1,A}\left(3_{3}\right)=P_{B2,A}\left(3_{3}\right)=1/9.$$

Here, the subscript *N* denotes that no box is opened by Bob. Hence, the probability that Alice detects the ball in box 3 is not changed by Bob making a measurement. Maroney referred to such a measurement as *operationally non-disturbing* [9] since Alice cannot detect Bob’s interference if she is restricted to opening box 3. This is referred to as Condition 1 in Maroney’s paper [9].

On the other hand, if Bob makes a measurement, then the final state on average after Alice’s transformations will be the mixture

(16) $$\begin{array}{ccc} \rho_{mix,m} & = & \frac{1}{6}U_{f}\left|1\rangle \langle 1\right|U_{f}^{†}+\frac{2}{6}U_{f}\left(\frac{|2\rangle +|3\rangle }{\sqrt{2}}\right)\left(\frac{\langle 2|+\langle 3|}{\sqrt{2}}\right)U_{f}^{†}\\ & & +\frac{1}{6}U_{f}\left|2\rangle \langle 2\right|U_{f}^{†}+\frac{2}{6}U_{f}\left(\frac{|1\rangle +|3\rangle }{\sqrt{2}}\right)\left(\frac{\langle 1|+\langle 3|}{\sqrt{2}}\right)U_{f}^{†}. \end{array}$$

The relative probabilities for Alice detecting the ball in box 1, 2 or 3 if Bob makes a measurement are 1/3, 5/9, and 1/9, compared to 0, 8/9, and 1/9 given by (14), if Bob makes no measurement. We see that, overall, the probabilities are changed on Bob’s measurement.

### Experimental Realization

The experimental realization requires the measurement of the probabilities as predicted above for the paradox. In particular, we require to show condition (13). In a real experiment, where there will be nonideal outcomes, the paradox would appear if we can demonstrate

(17) $$P_{B1,A}\left(1_{2}|3_{3}\right)+P_{B2,A}\left(2_{2}|3_{3}\right)>1.$$

This is confirmed by Maroney [9], who transformed the condition for the paradox into the condition to violate a Leggett–Garg inequality, as we will summarize in Section 6.

## 3. Mesoscopic Paradox

A macroscopic version of the paradox can be constructed by considering that the three states |1〉, |2〉, and |3〉 become macroscopically distinct. It is also necessary to identify suitable unitary transformations. Macroscopic and mesoscopic versions can be constructed in a number of ways. In this section, we consider a mesoscopic example that is a direct mapping of the original three-box paradox, with the generalization that the “particle” comprises *N* quanta. In Section 4, we consider a macroscopic example involving coherent states of a single-mode field, which allows a greater depth of study of the dynamics associated with the unitary operations.

### Number States

We analyze a proposal that maps directly onto the original paradox described in Section 2, where the three boxes correspond to distinct modes. We let

(18) $$\begin{array}{ccc} |1\rangle & = & {|N\rangle }_{1}{|0\rangle }_{2}{|0\rangle }_{3}\\ |2\rangle & = & {|0\rangle }_{1}{|N\rangle }_{2}{|0\rangle }_{3}\\ |3\rangle & = & {|0\rangle }_{1}{|0\rangle }_{2}{|N\rangle }_{3} \end{array}$$

where |N〉 is a number (Fock) state, the eigenstate of the boson number operator $\hat{n}$. The number state ${|n\rangle }_{i}$ of the *i*-th mode is denoted by the subscript i=1,2,3. The subscript is omitted where the meaning is clear. The modes are prepared in the superposition state

(19) $$|\psi_{sup}\rangle =\frac{e^{i\varphi }}{\sqrt{3}}(|3\rangle +e^{i\varphi_{1}}(i|2\rangle -\left|1\rangle )\right)$$

where φ and $\varphi_{1}$ are phase shifts, and the modes may be spatially separated. These states are tripartite extensions of NOON states [29].

The unitary transformations necessary for the three-box paradox are achieved by an interaction $H_{kl}$ that transforms the state ${|N\rangle }_{k}{|0\rangle }_{l}$ into the superposition [26,30,31,32]

(20) $$e^{i\varphi (\theta )}{(\cos\theta |N\rangle }_{k}{|0\rangle }_{l}-{i\sin\theta |0\rangle }_{k}{|N\rangle }_{l})$$

and that the state ${|0\rangle }_{k}{|N\rangle }_{l}$ into

(21) $$e^{i\varphi^{\prime }\left(\theta \right)}{(\sin\theta |N\rangle }_{k}{|0\rangle }_{l}+{i\cos\theta |0\rangle }_{k}{|N\rangle }_{l}).$$

**Figure 1:**
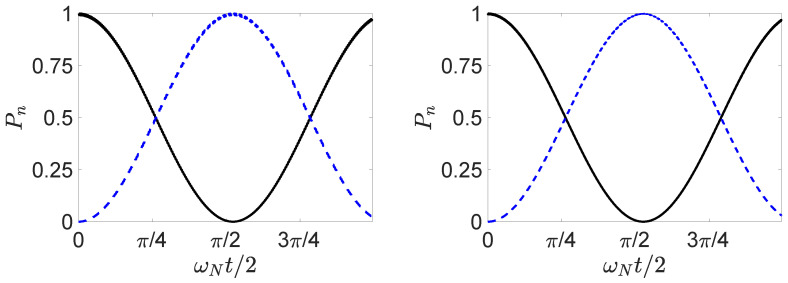
Realization of a nonlinear beam splitter: solutions are shown for the Hamiltonian $H_{kl}$ after a time *t* with initial state ${|N\rangle }_{k}{|0\rangle }_{l}$. Here N=2, κ=1, and g=30 (**left**), and N=5, κ=20, and g=333.33 (**right**). $P_{N}$ (black solid line) is the probability for all *N* bosons to be in mode *k*; $P_{0}$ (blue dashed line) is the probability for all *N* bosons to be in mode *l*. The parameters identify regimes optimal, or nearly optimal, for the nonlinear beam splitter interaction, where $P_{N}+P_{0}∼1$ and $P_{N}∼\cos^{2}\omega_{N}t$.

For N=1, this is achieved by beam splitters or polarizing beam splitters. For N≥1, we use the Josephson interaction that couples modes *k* and *l*, given as

(22) $$H_{kl}=\kappa \left({\hat{a}}_{k}^{†}{\hat{a}}_{l}+{\hat{a}}_{k}{\hat{a}}_{l}^{†}\right)+g{\hat{a}}_{k}^{†2}{\hat{a}}_{k}^{2}+g{\hat{a}}_{l}^{†2}{\hat{a}}_{l}^{2}$$

so that $U=e^{-iH_{kl}t/\hbar }$. Calculations have shown the result (20) to be realized to an excellent approximation, for optimised parameters and for N≲100, to the extent that Bell violations are predicted for systems where the spin states |↑〉 and |↓〉 become the mesoscopically distinct states ${|N\rangle }_{k}{|0\rangle }_{l}$ and ${|0\rangle }_{k}{|N\rangle }_{l}$ [26]. Here, ${\hat{a}}_{k}$, ${\hat{a}}_{l}$ are the boson destruction operators for two field modes *k* and *l*, and κ and *g* are the interaction constants. The θ is a function of the interaction time *t* and can be selected so that 0≤θ≤2π. We introduce a scaled time $\theta =\omega_{N}t$, where solutions are given in [32]. Here, we determine θ numerically by solving for the time $T_{NOON}$ taken for the system to evolve from ${|N\rangle }_{k}{|0\rangle }_{l}$ to (20) where θ=π/4. The solutions illustrating (20) are shown in Figure 1.

The state $|\psi_{sup}\rangle$ is created from state |3〉 using the interaction $H_{kl}$ as follows. We define $U_{f}$ so that

(23) $$\begin{array}{ccc} U_{f}|\psi_{f}\rangle & = & |3\rangle \\ U_{f}^{-1}|3\rangle & = & |\psi_{f}\rangle \end{array}$$

where $|\psi_{f}\rangle$ will be the post-selected state. First, we examine how to create the initial superposition state $|\psi_{sup}\rangle$ from |3〉, i.e., we find $U_{i}$ such that

(24) $$U_{i}\left|3\rangle =\right|\psi_{sup}\rangle .$$

The state

(25) $$|\psi_{2i}\rangle =e^{i\varphi }(\frac{1}{\sqrt{3}}|3\rangle +i\sqrt{\frac{2}{3}}\left|2\rangle \right)$$

is first created from the initial state |3〉 by evolving with $H_{32}$ for a suitable time $t_{1i}$, given by $\theta =\omega_{N}t_{1i}=\cos^{-1}\left(1/\sqrt{3}\right)$ where 3π/2<θ<2π. We find $U_{1i}|3\rangle =e^{-iH_{32}t_{1i}/\hbar }|3\rangle$ where

(26) $$U_{1i}=\frac{1}{\sqrt{3}}\left(\begin{array}{ccc} \sqrt{3} & 0 & 0\\ 0 & -ie^{i\varphi } & ie^{i\varphi }\sqrt{2}\\ 0 & e^{i\varphi }\sqrt{2} & e^{i\varphi } \end{array}\right).$$

Then, the interaction $H_{21}$ for the time $t_{2i}$ given by $\theta =\omega_{N}t_{2}=7\pi /4$ transforms $|2\rangle \to \frac{e^{i\varphi_{1}}}{\sqrt{2}}(|2\rangle +i|1\rangle )$, to give

(27) $$|\psi_{sup}\rangle =\frac{e^{i\varphi }}{\sqrt{3}}(|3\rangle +e^{i\varphi_{1}}(i|2\rangle -|1\rangle ).$$

We find $U_{2i}|\psi_{2i}\rangle =e^{-iH_{21}t_{2i}/\hbar }|\psi_{2i}\rangle$, where

(28) $$U_{2i}=\frac{1}{\sqrt{2}}\left(\begin{array}{ccc} -ie^{i\varphi_{1}} & ie^{i\varphi_{1}} & 0\\ e^{i\varphi_{1}} & e^{i\varphi_{1}} & 0\\ 0 & 0 & \sqrt{2} \end{array}\right).$$

We find

(29) $$\begin{array}{ccc} U_{i} & = & U_{2i}U_{1i}\\ & = & \frac{1}{\sqrt{6}}\left(\begin{array}{ccc} -i\sqrt{3}e^{i\varphi_{1}} & e^{i\varphi_{1}}e^{i\varphi } & i\sqrt{2}e^{i\varphi_{1}}e^{i\varphi }\\ e^{i\varphi_{1}}\sqrt{3} & -ie^{i\varphi_{1}}e^{i\varphi } & ie^{i\varphi_{1}}\sqrt{2}e^{i\varphi }\\ 0 & 2e^{i\varphi } & \sqrt{2}e^{i\varphi } \end{array}\right) \end{array}$$

and hence

(30) $$U_{i}^{†}=\frac{1}{\sqrt{6}}\left(\begin{array}{ccc} i\sqrt{3}e^{-i\varphi_{1}} & e^{-i\varphi_{1}}\sqrt{3} & 0\\ e^{-i\varphi_{1}}e^{-i\varphi } & ie^{-i\varphi }e^{-i\varphi_{1}} & 2e^{-i\varphi }\\ -\sqrt{2}e^{-i\varphi_{1}}e^{-i\varphi } & -i\sqrt{2}e^{-i\varphi_{1}}e^{-i\varphi } & \sqrt{2}e^{-i\varphi } \end{array}\right).$$

**Figure 2:**
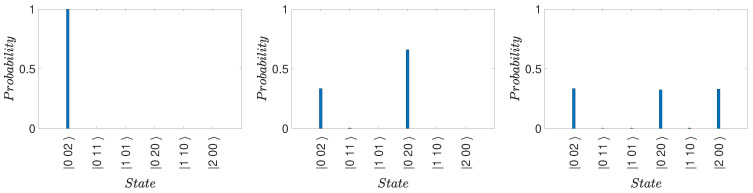
Creation of the superposition $|\psi_{sup}\rangle$ from |3〉, for N=2. The values of κ and *g* are chosen as in Figure 1. Each sequence shows the initial state |3〉 (**left**), the intermediate state $U_{1i}|3\rangle$ (**center**), and the final state $U_{2i}U_{1i}|3\rangle$ (**right**), where $U_{1i}=e^{-iH_{32}t_{1i}/\hbar }$ and $U_{2i}=e^{-iH_{21}t_{2i}/\hbar }$ for suitable choices of times $t_{1i}$ and $t_{2i}$. The probability that the system is in state $|k,l,m\rangle \equiv {|k\rangle }_{1}{|l\rangle }_{2}{|m\rangle }_{3}$ is depicted at the given time in the sequence. The probability that the system is in a state different to |1〉, |2〉, or |3〉 is less than $3\times 10^{-3}$.

The dynamics arising from the Hamiltonians $H_{32}$ and $H_{21}$ for actual values of *g* and κ does not in general constrain the system to the states |1〉, |2〉, or |3〉. Full solutions are depicted in Figure 2. For the parameters given, the probability that the system is found in a state different to |1〉, |2〉, or |3〉 is, however, negligible.

Now reversing, we see that the post-selected state

(31) $$|\psi_{f}\rangle =\frac{e^{i\varphi }}{\sqrt{3}}(-|3\rangle +e^{i\varphi_{1}}(i|2\rangle -\left|1\rangle )\right)$$

can be created from |3〉 using $U_{f}^{-1}$, by applying $H_{32}$ with $\theta =\omega_{N}t$, so that $\cos\theta =-\frac{1}{\sqrt{3}}$, $\sin\theta =-\sqrt{2/3}$, followed by $H_{21}$. We start with |3〉 and act with $U_{1f}=U_{32}=e^{-iH_{32}t/\hbar }$, for $t=\theta /\omega_{N}$, where

(32) $$U_{1f}=\frac{1}{\sqrt{3}}\left(\begin{array}{ccc} \sqrt{3} & 0 & 0\\ 0 & -ie^{i\varphi } & ie^{i\varphi }\sqrt{2}\\ 0 & -e^{i\varphi }\sqrt{2} & -e^{i\varphi } \end{array}\right).$$

Here,

$$U_{1f}^{†}=\frac{1}{\sqrt{3}}\left(\begin{array}{ccc} \sqrt{3} & 0 & 0\\ 0 & ie^{-i\varphi } & -e^{-i\varphi }\sqrt{2}\\ 0 & -ie^{-i\varphi }\sqrt{2} & -e^{-i\varphi } \end{array}\right).$$

This creates state $|\psi_{1f}\rangle =\frac{e^{i\varphi }}{\sqrt{3}}(-|3\rangle +i\sqrt{2}\left|2\rangle \right)$. Then, we act on $|\psi_{1f}\rangle$ with $U_{2f}=U_{21}=e^{-iH_{21}t/\hbar }$ for *t* such that $t=7\pi /4\omega_{N}$ so that $U_{2f}=U_{2i}$. We see that

(33) $$\begin{array}{ccc} U_{f}^{-1} & = & U_{2f}U_{1f}\\ & = & \frac{1}{\sqrt{6}}\left(\begin{array}{ccc} -i\sqrt{3}e^{i\varphi_{1}} & e^{i\varphi_{1}}e^{i\varphi } & -e^{i\varphi_{1}}e^{i\varphi }\sqrt{2}\\ \sqrt{3}e^{i\varphi_{1}} & -ie^{i\varphi_{1}}e^{i\varphi } & ie^{i\varphi }e^{i\varphi_{1}}\sqrt{2}\\ 0 & -2e^{i\varphi } & -\sqrt{2}e^{i\varphi } \end{array}\right). \end{array}$$

This gives state $|\psi_{f}\rangle$. Hence, $U_{f}^{-1}\left|3\rangle =\right|\psi_{f}\rangle$ where $U_{f}^{-1}=U_{2f}U_{1f}$. Hence, $U_{f}=U_{1f}^{†}U_{2f}^{†}$. Hence, Alice’s measurements are $U_{2f}^{-1}$ followed by $U_{1f}^{-1}$. This gives $U_{f}|\psi_{f}\rangle =|3\rangle$. We find

(34) $$\begin{array}{ccc} U_{f} & = & \frac{1}{\sqrt{6}}\left(\begin{array}{ccc} i\sqrt{3}e^{-i\varphi_{1}} & \sqrt{3}e^{-i\varphi_{1}} & 0\\ e^{-i\varphi_{1}}e^{-i\varphi } & ie^{-i\varphi_{1}}e^{-i\varphi } & -2e^{-i\varphi }\\ -e^{-i\varphi_{1}}e^{-i\varphi }\sqrt{2} & -ie^{-i\varphi }e^{-i\varphi_{1}}\sqrt{2} & -\sqrt{2}e^{-i\varphi } \end{array}\right). \end{array}$$

**Figure 3:**
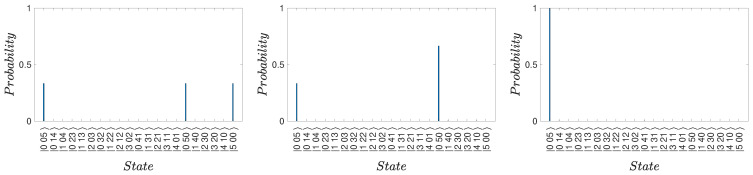
Creation of the superposition |3〉 from the post-selected state $|\psi_{f}\rangle$ using the operations $U_{f}$, for N=5. The values of κ and *g* are chosen as in Figure 1. Each sequence shows the initial state $|\psi_{f}\rangle$ (**left**), the intermediate state $U_{2f}^{-1}|\psi_{f}\rangle$ (**center**), and the final state $U_{1f}^{-1}U_{2f}^{-1}|\psi_{f}\rangle$ (**right**), where $U_{1f}\equiv U_{32}$ and $U_{2f}\equiv U_{21}$, as defined in the text. The probability that the system is in state $|k,l,m\rangle \equiv {|k\rangle }_{1}{|l\rangle }_{2}{|m\rangle }_{3}$ is depicted at the given time in the sequence. The probability that the system is in a state different to |1〉, |2〉, or |3〉 is less than $6\times 10^{-3}$.

Full solutions are depicted in Figure 3.

The paradox follows as for the original paradox. Bob determines whether the system is in state |1〉 or not. Alternatively, he determines whether the system is in state |2〉 or not. If after Bob’s measurements the system is in state |1〉, then after Alice’s unitary “shuffling” operations the system is in

(35) $$U_{f}|1\rangle =\frac{e^{-i\varphi_{1}}}{\sqrt{6}}\left(\begin{array}{c} i\sqrt{3}\\ e^{-i\varphi }\\ -e^{-i\varphi }\sqrt{2} \end{array}\right).$$

**Figure 4:**
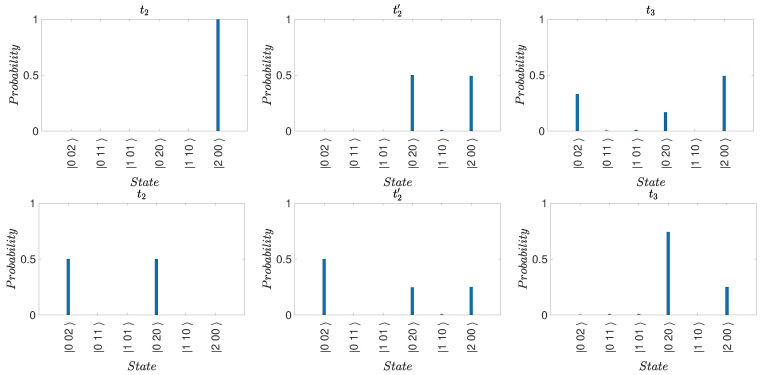
The dynamics corresponding to Alice’s transformations $U_{f}$ if (**top row**) Bob detects *N* photons in box 1 at time $t_{2}$, or (**lower row**) if Bob detects the photons are not in box 1 at time $t_{2}$. The histograms give the probabilities for detecting *N* photons in box *K*. The probability that the system is in state $|k,l,m\rangle \equiv {|k\rangle }_{1}{|l\rangle }_{2}{|m\rangle }_{3}$ is depicted at the given time in the sequence. Here, we show the initial state after Bob’s measurement at time $t_{2}$ (**left**), the state generated at time $t_{2}^{\prime }$ after Alice’s transformation $U_{2f}^{-1}$ (**center**), and the state generated at time $t_{3}$ after Alice’s further transformation $U_{1f}^{-1}$ (**right**). The final state after Alice’s total transformation $U_{f}$ is (35) (top) or (37) (lower) to an excellent approximation. The probability that the system is in any other state apart from |00N〉, |0N0〉 or |N00〉 is less than $8\times 10^{-3}$. The solutions are for N=2.

The solutions in Figure 4 for the optimal choice of κ and *g* give agreement for N=2. Similarly, if Bob determines that the system is in state |2〉, then after Alice’s unitary operations the system is in

(36) $$U_{f}|2\rangle =\frac{e^{-i\varphi_{1}}}{\sqrt{6}}\left(\begin{array}{c} \sqrt{3}\\ ie^{-i\varphi }\\ -ie^{-i\varphi }\sqrt{2} \end{array}\right).$$

If Bob determines the system is not in |1〉, then at time $t_{2}$ the system is in $(|3\rangle +e^{i\varphi_{1}}i|2\rangle )/\sqrt{2}$. The final state after Alice’s transformations is

(37) $$\begin{array}{ccc} U_{f}\frac{1}{\sqrt{2}}\left(\begin{array}{c} 0\\ ie^{i\varphi_{1}}\\ 1 \end{array}\right) & = & \frac{1}{2\sqrt{3}}\left(\begin{array}{c} \sqrt{3}\\ -3e^{-i\varphi }\\ 0 \end{array}\right). \end{array}$$

This is depicted in Figure 4. If Bob determines the system is not in |2〉, then at time $t_{2}$ the system is in $(|3\rangle -e^{i\varphi_{1}}\left|1\rangle \right)/\sqrt{2}$. The final state after Alice’s transformations is

(38) $$\begin{array}{ccc} U_{f}\frac{1}{\sqrt{2}}\left(\begin{array}{c} -e^{i\varphi_{1}}\\ 0\\ 1 \end{array}\right) & = & \frac{1}{2\sqrt{3}}\left(\begin{array}{c} -i\sqrt{3}\\ -3e^{-i\varphi }\\ 0 \end{array}\right). \end{array}$$

The paradox occurs because Alice finds that there is zero probability of finding the system in the state |3〉 in both cases. If after her transformations she finds the system to be in state |3〉, then she knows for certain that Bob detected the ball in the box he opened.

The calculations for the marginal and joint probabilities follow as in Section 2 for the original paradox, to give identical results, with the prediction of (13). An experimental realization of the paradox would verify these probabilities, in particular Condition (17).

Similarly, the aspect of measurement disturbance can be studied, to give results as in Section 2, with the same conclusions. We note that if there is no measurement by Bob, then the final state at time $t_{3}$ is

(39) $$\begin{array}{ccc} U_{f}|\psi_{sup}\rangle & = & \frac{1}{3\sqrt{2}}\left(\begin{array}{c} 0\\ -4\\ \sqrt{2} \end{array}\right). \end{array}$$

This implies that $P_{N,A}\left(3_{3}\right)=1/9$. As for the original paradox, this agrees with the value of $P_{B1,A}\left(3_{3}\right)=1/9$, calculated from the above results, where Bob opens box 1. Similarly, $P_{B2,A}\left(3_{3}\right)=1/9$. Hence, condition (15) holds. The probability that Alice detects the ball in box 3 is not changed by Bob making a measurement. The measurement is operationally non-disturbing [9] since Alice cannot detect Bob’s interference if she is restricted to opening box 3. Condition 1 of the paper by Maroney [9] is satisfied.

On the other hand, if Bob makes a measurement, the overall system reduces to the mixture corresponding to the outcomes obtained by Bob. If he opens box 1, the state is

(40) $$\begin{array}{ccc} \rho_{mix,1}\left(t_{3}\right) & = & \frac{1}{3}U_{f}\left|1\rangle \langle 1\right|U_{f}^{†}\\ & & +\frac{1}{3}U_{f}(|3\rangle +e^{i\varphi_{1}}i|2\rangle )(\langle 3|-ie^{-i\varphi_{1}}\langle 2|)U_{f}^{†}. \end{array}$$

Similarly, if he opens box 2, the state is

(41) $$\begin{array}{ccc} \rho_{mix,2}\left(t_{3}\right) & = & \frac{1}{3}U_{f}\left|2\rangle \langle 2\right|U_{f}^{†}\\ & & +\frac{1}{3}U_{f}(|3\rangle -e^{i\varphi_{1}}\left|1\rangle )(\langle 3\right|-e^{i\varphi_{1}}\langle 1|)U_{f}^{†}. \end{array}$$

This leads to a detectable change in the measured probabilities. As for the original example in Section 2, the relative probabilities for Alice detecting the ball in box 1, 2, or 3 if Bob makes a measurement are different to those if Bob makes no measurement.

We emphasize that the solution (20) for the evolution given by *H* is approximate. Actual solutions are given in the figures and are sufficient to confirm the three-box paradox for moderate *N*, illustrated by N=2 and N=5.

## 4. Macroscopic “Three-box” Paradox with Cat States

The example of Section 3 considered states distinct by *N* quanta. However, the calculations involved the interaction $H_{kl}$, which was solved for N∼5. One way to achieve a more macroscopic realization of the three-box paradox is to consider the coherent states |α〉 of a single-mode field. In this section, we propose such a paradox, where the separation between the relevant coherent states can be made arbitrarily large. In order to achieve a feasible realization, the macroscopic paradox is based on a modified version of the original three-box paradox.

Superpositions of macroscopically distinct coherent sates are referred to as “cat states” [24,33,34,35]. Such states can be generated in optical cavities with dissipation and/or using conditional measurements [18,19,35,36,37,38,39,40,41,42,43,44]. Here, we take a simple model in which the cat states are created from a nonlinear dispersive medium, where losses are assumed to be minimal [34,45]. The unitary operations are solved analytically and have been realized experimentally, to create cat states for large α [18,46].

### 4.1. Coherent-State Model: k>2

We propose four states defined as

(42) $$\begin{array}{ccc} |1\rangle & = & |-i\alpha_{0}\rangle \\ |2\rangle & = & |i\alpha_{0}\rangle \\ |3\rangle & = & |\alpha_{0}\rangle \\ |4\rangle & = & |-\alpha_{0}\rangle \end{array}$$

where |α〉 is a coherent state of a single-mode field. As $\alpha_{0}\to \infty$, these states become orthogonal. We note that for sufficiently large $\alpha_{0}$, the states can be distinguished by simultaneous quadrature phase amplitude measurements $\hat{X}=\left(\hat{a}+{\hat{a}}^{†}\right)/\sqrt{2}$ and $\hat{P}=\left(\hat{a}-{\hat{a}}^{†}\right)/\sqrt{2}i$, where $\hat{a}$ and ${\hat{a}}^{†}$ are the field boson destruction and creation operators [34].

The system can be prepared at time $t_{1}$ from the state |3〉 in the superposition [27,34] of type (1), by applying a set of unitary transformations based on nonlinear interactions. Following the work of Yurke and Stoler [34], we consider the evolution of a single mode system prepared in a coherent state under the influence of a nonlinear Hamiltonian written in the Schrödinger picture as,

(43) $$H_{NL}=\omega \hat{n}+\Omega {\hat{n}}^{k}$$

where ω gives the harmonic oscillator angular frequency, Ω represents the strength of the nonlinear term, and $\hat{n}={\hat{a}}^{†}\hat{a}$ is the field number operator. We have taken ℏ=1. Here, *k* is a positive integer. After an interaction time of $t=\frac{\pi }{2\Omega }$, the system with k>2 initially prepared in a coherent state |α〉 becomes

(44) $$\begin{array}{ccc} |\psi_{sup}\rangle & \equiv & |\psi_{1}\rangle =\frac{1}{2}(|1\rangle -|2\rangle +|3\rangle +\left|4\rangle \right)\\ & = & \frac{1}{2}(|-i\alpha_{0}\rangle -|i\alpha_{0}\rangle +|\alpha_{0}\rangle +|-\alpha_{0}\rangle ) \end{array}$$

which for large $\alpha_{0}$ is a four-component cat state. We can interpret the creation of the state as a transformation using a unitary operator $U_{i}=U\left(\frac{\pi }{2\Omega }\right)$ given by

(45) $$U_{i}|3\rangle =U_{i}|\alpha_{0}\rangle =|\psi_{sup}\rangle$$

where $U\left(\frac{\pi }{2\Omega }\right)=e^{-i\Omega {\hat{n}}^{k}t}=e^{-i\pi {\hat{n}}^{k}/2}$ with ℏ=1. For large $\alpha_{0}$ where the four states can form a basis set {|1〉,|2〉, |3〉, |4〉}, it is convenient to identify the transformation as a matrix

(46) $$\begin{array}{ccc} U_{i} & = & \frac{1}{2}\left(\begin{array}{cccc} 1 & 1 & 1 & -1\\ 1 & 1 & -1 & 1\\ -1 & 1 & 1 & 1\\ 1 & -1 & 1 & 1 \end{array}\right) \end{array}$$

where the basis states |k〉 correspond to column matrices $\left(a_{j1}\right)$ with coefficients given as $a_{j1}=\delta_{jk}$. It is straightforward to verify that the operations $U_{i}|k\rangle$ realize the correct final states.

To account for the four states, we consider a modified version of the three-box paradox. Bob can consider to determine whether the system is in one of the four states |k〉, analogous to four boxes. The system that Bob measures is in the state $|\psi_{sup}\rangle =|1\rangle -|2\rangle +|3\rangle +|4\rangle$. Bob may make measurements to determine whether the system is in one of the states |2〉 or |4〉, or not. If the system can be determined to be in neither |2〉 nor |4〉, then the reduced state for the system is |1〉+|3〉. Alternatively, Bob may make measurements to determine whether the system is in one of the states |1〉 or |4〉, or not. If the system is determined to be in neither |1〉 nor |4〉, then the reduced state for the system is −|2〉+|3〉.

Suppose that Alice post-selects for the state

(47) $$\begin{array}{ccc} |\psi_{f}\rangle & = & (-|1\rangle +|2\rangle +|3\rangle +|4\rangle )/2\\ & = & \frac{1}{2}(|\alpha_{0}\rangle +|-\alpha_{0}\rangle -|-i\alpha_{0}\rangle +|i\alpha_{0}\rangle ) \end{array}$$

which is generated from $|\alpha_{0}\rangle =|3\rangle$ by a transformation

(48) $$U_{f}^{-1}\left|3\rangle =\right|\psi_{f}\rangle .$$

**Figure 5:**
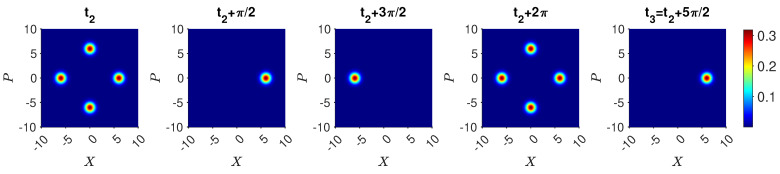
Dynamics of Alice’s transformation $U_{f}$ performed on the system in state $|\psi_{f}\rangle =-|1\rangle +|2\rangle +|3\rangle +|4\rangle$ at time $t_{2}$. The operation $U_{f1}$ is carried out by evolving under $H_{NL}$ for a time t=3π/2Ω. Next, the operation $U_{f2}$ is carried out by evolving under $H_{NL}$ for a further time t=π/Ω. At time $t_{3}$ after the combined operation $U_{f}=U_{f2}U_{f1}$, the system is in state |3〉.

The dynamics for $U_{f}$ are shown in Figure 5. The inverse is achieved by first transforming $|\alpha_{0}\rangle \to$$|-\alpha_{0}\rangle$ using the nonlinear interaction modeled by the Hamiltonian $H_{NL}$ of (43). Defining $U_{f2}=U\left(\frac{\pi }{\Omega }\right)=e^{-i\Omega {\hat{n}}^{k}t}=e^{-i\pi {\hat{n}}^{k}}$ where the interaction time is t=π/Ω, the state $|\alpha_{0}\rangle$ becomes $|-\alpha_{0}\rangle$ for all integers *k*. We identify

(49) $$\begin{array}{ccc} U_{f2}^{-1} & = & \left(\begin{array}{cccc} 0 & 1 & 0 & 0\\ 1 & 0 & 0 & 0\\ 0 & 0 & 0 & 1\\ 0 & 0 & 1 & 0 \end{array}\right) \end{array}$$

and note that $U_{f2}^{-1}|\alpha_{0}\rangle =|-\alpha_{0}\rangle$. We also see that $U_{f2}^{-1}=U_{f2}$. The second stage of the generation is to transform $|4\rangle =|-\alpha_{0}\rangle$ by the unitary transformation $U_{f1}^{-1}$ so that

(50) $$U_{f1}^{-1}\left|4\rangle =\right|\psi_{f}\rangle .$$

We find that $U_{f1}^{-1}=U\left(\frac{\pi }{2\Omega }\right)=e^{-i\Omega {\hat{n}}^{k}t}$ where t=π/2Ω and note that $U_{f1}^{-1}=U_{i}$. It is straightforward to verify that $U_{f1}^{-1}\left|4\rangle =\right|\psi_{f}\rangle$. In fact, $U_{f1}=U_{i}^{-1}$. Since for any unitary interaction $U=e^{-iHt}$, $U^{-1}=U^{†}$, the inverse $U^{-1}$ corresponds to $U^{-1}=e^{iHt}$. Since the evolution is periodic, with period 2π/Ω [34], this corresponds to $U^{-1}=e^{iH_{NL}\left(t-2\pi /\Omega \right)}=e^{-iH_{NL}\left(2\pi /\Omega -t\right)}$, and we find that $U_{f1}=U_{i}^{-1}=U\left(\frac{3\pi }{2\Omega }\right)$. The $U_{f1}$ is realized by Alice evolving the system forward in time by the amount t=3π/2Ω. We may express this as

(51) $$U_{f1}^{-1}U_{f2}^{-1}\left|3\rangle =\right|\psi_{f}\rangle$$

which implies

(52) $$U_{f}|\psi_{f}\rangle \equiv U_{f2}U_{f1}|\psi_{f}\rangle =|3\rangle .$$

The operations $U_{f}$ correspond to Alice first evolving the system under *H* with k>2 for a time t=3π/2Ω and then applying the evolution *H* for a time t=π/Ω. We find that $U_{f}=U_{f2}U_{f1}=U_{f2}^{-1}U_{i}^{-1}$: hence, in matrix form

(53) $$\begin{array}{ccc} U_{f} & = & \frac{1}{2}\left(\begin{array}{cccc} 1 & 1 & 1 & -1\\ 1 & 1 & -1 & 1\\ -1 & 1 & 1 & 1\\ 1 & -1 & 1 & 1 \end{array}\right). \end{array}$$

Figure 5 shows the dynamics of Alice’s $U_{f}$, where the system is initially in the state $|\psi_{f}\rangle$. The evolution confirms that the final state is indeed |3〉.

The paradox is realized when Alice performs the measurement given by the transformation $U_{f}$ and detects whether the system is in state |3〉. We check for the paradox as follows. If Bob detects the system to be in |2〉, then the evolution by Alice gives

(54) $$\begin{array}{ccc} U_{f}|2\rangle & = & \frac{1}{2}\left(\begin{array}{c} 1\\ 1\\ 1\\ -1 \end{array}\right). \end{array}$$

If Bob detects that the system is in state |4〉, then the evolution by Alice gives

(55) $$\begin{array}{ccc} U_{f}|4\rangle & = & \frac{1}{2}\left(\begin{array}{c} -1\\ 1\\ 1\\ 1 \end{array}\right). \end{array}$$

If Bob detects that the system is in state |1〉, then the evolution by Alice gives

(56) $$\begin{array}{ccc} U_{f}|1\rangle & = & \frac{1}{2}\left(\begin{array}{c} 1\\ 1\\ -1\\ 1 \end{array}\right). \end{array}$$

If Bob detects that the system is not in |2〉 or |4〉, then the system is in the reduced state |1〉+|3〉, and the evolution by Alice gives the final state

(57) $$\begin{array}{ccc} U_{f}(|1\rangle +\left|3\rangle \right)/\sqrt{2} & = & \frac{1}{\sqrt{2}}\left(\begin{array}{c} 1\\ 0\\ 0\\ 1 \end{array}\right). \end{array}$$

If Bob detected that the system is not in |1〉 or |4〉, then the system is in reduced state −|2〉+|3〉, and the evolution by Alice gives the final state

(58) $$\begin{array}{ccc} U_{f}(-|2\rangle +\left|3\rangle \right)/\sqrt{2} & = & \frac{1}{\sqrt{2}}\left(\begin{array}{c} 0\\ -1\\ 0\\ 1 \end{array}\right). \end{array}$$

**Figure 6:**
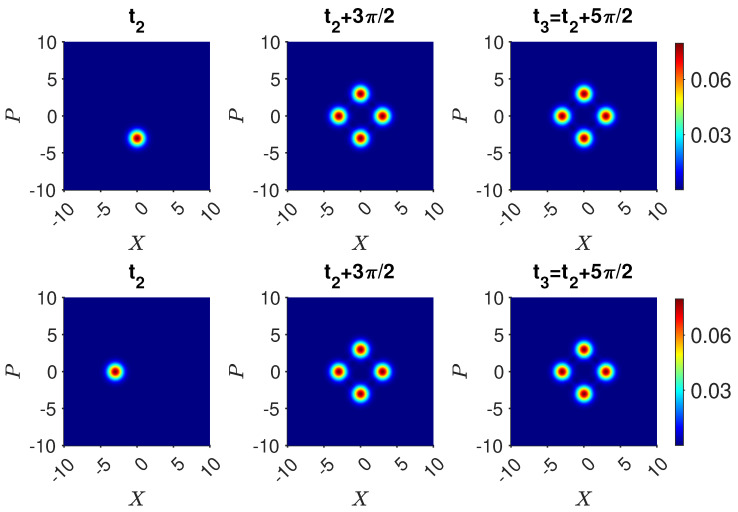
Dynamics of Alice’s transformation $U_{f}$ performed at time $t_{2}$ after Bob makes his measurement. Here, we take the case where Bob obtains the following results: (**top**) that the ball is in box 1 (implying the system is in state |1〉 at time $t_{2}$) or (**lower**) that the ball is in box 4 (implying the system is in state |4〉 at time $t_{2}$). The figures show contour plots of Q(α) for $\alpha_{0}=3$ and k=3. Here, $U_{f}=U_{f2}U_{f1}$. The plots show the state after the first transformation $U_{f1}$ at time $t_{2}+3\pi /2$. In both cases, there is a finite probability of Alice finding the ball in box 3.

**Figure 7:**
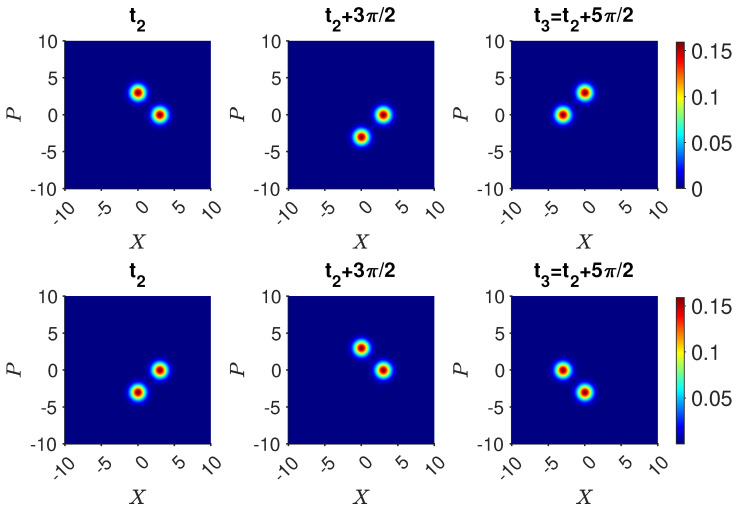
Dynamics of Alice’s transformation $U_{f}$ performed at time $t_{2}$ after Bob makes his measurement. Here, we take the case where Bob obtains the following results: (**top**) that the ball is not in box 1 or 4 (implying the system in state −|2〉+|3〉 at time $t_{2}$) or (**lower**) that the ball is not in box 2 or 4 (so that the system in state |1〉+|3〉 at time $t_{2}$). The figures show contour plots of Q(α) for $\alpha_{0}=3$ and k=3. In both cases, there is zero probability of Alice finding the ball in box 3.

In both cases, there is zero probability of Alice measuring the system to be in state |3〉=|α〉. Hence, if Alice detects the system to be in $|\alpha_{0}\rangle$ at time $t_{3}$, she knows that Bob detected the ball to be in one of the boxes he opened. The dynamics is confirmed in Figure 6 and Figure 7. The figures plot the dynamical sequences where Bob detects the ball and where Bob detects no ball in boxes 1 or 4. We confirm that for the latter there is zero probability of the system being found by Alice in state |3〉.

The calculations can be summarized. If Bob opens box 1 (B1), then the probability of him detecting the ball is 1/4. Following [9], we write this as $P_{B1}\left(1_{2}\right)=1/4$. We note as in Section 2 that we label the time of completion of Bob’s measurement as $t_{2}$, whereas the time at which the state he is measuring has been prepared, just before his measurement, is denoted by $t_{1}$. The label choice makes no difference since $P_{BI}\left(1_{2}\right)=P_{BI}\left(1_{1}\right)$ and $P_{BI}\left(2_{2}\right)=P_{BI}\left(2_{1}\right)$. Continuing, we see also that $P_{B1,A}\left(3_{3}|1_{2}\right)=1/4$ and hence $P_{B1,A}\left(1_{2},3_{3}\right)=P_{B1,A}\left(3_{3}|1_{2}\right)P_{B1}\left(1_{2}\right)=1/16$. Similarly, denoting the probability of detecting the ball in either box 1 or 4 if both boxes are opened as $P_{B1,B4}\left(\left\{1_{2},4_{2}\right\}\right)$, we find $P_{B1,B2}\left(\left\{1_{2},4_{2}\right\}\right)=1/2$. From above, $P_{B1,A}\left(3_{3}|1_{2}\right)=1/4$, and similarly, $P_{B1,A}\left(3_{3}|1_{2}\right)=1/4$, but only one box can be detected with a ball. Hence, the probability of detecting a ball in box 3 at time $t_{3}$, given Bob detected a ball in either box 1 or 4 at time $t_{2}$, is $P_{B1,B4,A}\left(3_{3}|\left\{1_{2},4_{2}\right\}\right)=1/4$. Hence,

(59) $$\begin{array}{ccc} P_{B1,B4,A}\left(\left\{1_{2},4_{2}\right\},3_{3}\right) & = & P_{B1,B4,A}\left(3_{3}|\left\{1_{2},4_{2}\right\}\right)\\ & & \times P_{B1,B4}\left(\left\{1_{2},4_{2}\right\}\right)\\ & = & 1/8. \end{array}$$

The probability the ball is detected in box 3, if Bob opens boxes 1 and 4, is $P_{B1,B4,A}\left(3_{3}\right)=\frac{1}{2}\left\{\frac{1}{4}\right\}+\frac{1}{2}\left\{0\right\}=1/8$. Hence, we obtain the key result:

(60) $$P_{B1,B4,A}\left(\left\{1_{2},4_{2}\right\}|3_{3}\right)=1.$$

Similarly, $P_{B2,B4,A}\left(3_{3}\right)=1/8$, and

(61) $$P_{B2,B4,A}\left(\left\{2_{2},4_{2}\right\}|3_{3}\right)=1.$$

We also note that

(62) $$\begin{array}{ccc} P_{B1,B4,A}\left(4_{2},3_{3}\right) & = & P_{B1,B4}\left(3_{3}|4_{2}\right)P_{B1,B4}\left(4_{2}\right)\\ & = & 1/16 \end{array}$$

which implies since $P_{B1,B4,A}\left(3_{3}\right)=1/8$,

(63) $$P_{B1,B4,A}\left(4_{2}|3_{3}\right)=P_{B2,B4,A}\left(4_{2}|3_{3}\right)=1/2.$$

Hence, Alice knows that if she detects the ball in box 3, the ball was detected in box 4 only 50% of the time. Yet, we see that the ball was detected with certainty in the set of boxes (either 1 and 4, or 2 and 4) that Bob opened. The paradox is that it seems as though for 50% of the time, the ball would have had to have been detected in boxes 2 and 1, had that box been opened. An experimental realization would verify these probabilities. We obtain a paradox when

(64) $$\begin{array}{c} P_{B1,B4,A}\left(\left\{1_{2},4_{2}\right\}|3_{3}\right)+P_{B2,B4,A}\left(\left\{2_{2},4_{2}\right\}|3_{3}\right)\\ >1+P_{B1,B4,A}\left(4_{2}|3_{3}\right) \end{array}$$

assuming (63) is shown to hold. The paradox can be expressed as a violation of a Leggett–Garg inequality, in Section 6, and the condition derived using the approach of Maroney [9]. The violation of the Leggett–Garg inequality also gives a method for an experimental realization.

The effect of measurement disturbance is made apparent in this example. If Bob makes *no* measurement of the state prepared at time $t_{1}$, then the final state at time $t_{3}$ is $U_{f}|\psi_{sup}\rangle$:

(65) $$\begin{array}{ccc} U_{f}|\psi_{sup}\rangle & = & \left(\begin{array}{c} 0\\ 0\\ 0\\ 1 \end{array}\right). \end{array}$$

There is zero probability that Alice detects a ball in box 3: $P_{N,A}\left(3_{3}\right)=0$. Yet, from the results above, if Bob opens boxes 1 and 4, the probability of Alice observing the ball in box 3 is 1/8. Unlike the standard paradox, $P_{N,A}\left(3_{3}\right)\neq P_{B1,B4,A}\left(3_{3}\right)$. This means that Condition 1 of Ref. [9] is not satisfied. We note, however, that the probability of Alice detecting a ball in box 3, given that Bob makes a measurement, is not affected by which pair of boxes he opens: $P_{B1,B4,A}\left(3^{3}\right)=1/8$ and $P_{B2,B4,A}\left(3^{3}\right)=1/8$. This means part of Condition 1 of [9] is satisfied. The transformation $U_{f}$, equivalent to a shuffle, does not give a relative enhancement of the probability of Alice detecting a ball in box 3 depending on which boxes Bob opened.

### Experimental Realization

The experimental realization requires the measurement of the predicted probabilities of the paradox. In particular, we require to show condition (64), as we confirm in Section 6. The four states are not orthogonal except in the limit of infinite α. The states can be distinguished by simultaneous measurement of quadrature phase amplitudes $\hat{X}$ and $\hat{P}$, but for any finite α there is an overlap of the states, which introduces an error. For example, the states |α〉 and |−α〉 where α is real can be distinguished by measuring the sign of *x*, the outcome of $\hat{X}$, whether positive or negative. For the system in state |α〉, the distribution for *x* is a Gaussian function, $e^{-{\left(x-\sqrt{2}\alpha \right)}^{2}}/\pi^{1/2}$, with mean $\mu =\sqrt{2}\alpha$ and standard deviation $\sigma =1/\sqrt{2}$ [34]. There is always a nonzero probability for outcomes x<0. However, this probability becomes negligible for moderate α. For α=1, the probability that a system in |−α〉 can give a positive outcome for $\hat{X}$ is less than 3 percent. This error can be factored into the measurements of the probabilities used in the inequality (64). The ideal prediction corresponding to α→∞ gives the left side of the inequality as 2. This compares with the right side of the inequality, which is 1.5. The error due to the overlap of the coherent states will be negligible for experimentally achievable values of α>2 [18].

### 4.2. Coherent-State Model: k=2

The experimental realization of k>2 is, however, challenging. A promising similar but alternative proposal uses k=2, for which the unitary interactions described in Yurke and Stoler have been experimentally verified [18,46]. We define the states

(66) $$\begin{array}{ccc} |1\rangle & = & |-i\alpha_{0}\rangle \\ |2\rangle & = & |i\alpha_{0}\rangle \\ |3\rangle & = & e^{-i\pi /4}|\alpha_{0}\rangle \\ |4\rangle & = & e^{-i\pi /4}|-\alpha_{0}\rangle . \end{array}$$

We consider the state prepared at time $t_{1}$,

(67) $$|\psi_{sup}\rangle \equiv |\psi_{1}\rangle =\frac{1}{2}\{|1\rangle +|2\rangle +|3\rangle -|4\rangle \}.$$

This state [27,34]

(68) $$\begin{array}{c} |\psi_{sup}\rangle =\frac{1}{2}\{|-i\alpha_{0}\rangle +|i\alpha_{0}\rangle +e^{-i\pi /4}|\alpha_{0}\rangle -e^{-i\pi /4}|-\alpha_{0}\rangle \} \end{array}$$

is formed at time t=π/4Ω from $|\alpha_{0}\rangle$, using the evolution given by $H_{NL}$ of Equation (43) with k=2. We can interpret the creation of the state as a transformation using a unitary operator $U_{i}=U\left(\frac{\pi }{4\Omega }\right)$ given by

(69) $$U_{i}|3\rangle =U_{i}|\alpha_{0}\rangle =|\psi_{sup}\rangle$$

where $U\left(\frac{\pi }{4\Omega }\right)=e^{-i\Omega {\hat{n}}^{k}t}=e^{-i\pi {\hat{n}}^{k}/4}$ with ℏ=1.

Bob measures whether the system is in state |1〉 or |4〉, or not. If the system can be determined to be in neither |1〉 nor |4〉, then the reduced state for the system is |2〉+|3〉. Alternatively, Bob may make measurements to determine whether the system is in one of the states |2〉 or |4〉, or not. If the system is determined to be in neither |2〉 or |4〉, then the reduced state for the system is |1〉+|3〉.

Alice post-selects for the state

(70) $$\begin{array}{ccc} |\psi_{f}\rangle & = & \frac{1}{2}\{|1\rangle +|2\rangle -|3\rangle +\left|4\rangle \right\}\\ & = & \frac{1}{2}\{|-i\alpha_{0}\rangle +|i\alpha_{0}\rangle \\ & & -e^{-i\pi /4}|\alpha_{0}\rangle +e^{-i\pi /4}|-\alpha_{0}\rangle \} \end{array}$$

which is formed at time t=π/4Ω from $|-\alpha_{0}\rangle$, using k=2. We aim to find $U_{f}$ such that

(71) $$U_{f}^{-1}\left|3\rangle =\right|\psi_{f}\rangle .$$

As above, we first transform to |4〉. Defining $U_{f2}=U\left(\frac{\pi }{\Omega }\right)=e^{-i\Omega {\hat{n}}^{k}t}=e^{-i\pi {\hat{n}}^{k}}$ where the interaction time is t=π/Ω, the state $|\alpha_{0}\rangle$ becomes $|-\alpha_{0}\rangle$, for k=2. We see that $U_{f2}=U_{f2}^{-1}$. We then apply

(72) $$U_{f3}^{-1}U_{f2}^{-1}\left|3\rangle =\right|\psi_{f}\rangle$$

where we define $U_{f3}^{-1}=U\left(\frac{\pi }{4\Omega }\right)=e^{-i\Omega {\hat{n}}^{k}t}$ with interaction time as t=π/4Ω. We note that $U_{f3}=U\left(\frac{7\pi }{4\Omega }\right)=e^{-i\Omega {\hat{n}}^{k}t}$, which corresponds to the interaction time t=7π/4Ω. We find that $U_{f}=U_{f2}U_{f3}$. We find that $U_{f}=U_{f2}U_{f3}$.

Hence, Alice transforms the system according to $U_{f}$ and then detects whether the system is in state |3〉. The operations $U_{f}$ correspond to Alice first evolving the system under $H_{NL}$ with k=2 for a time t=7π/4Ω and then applying the evolution *H* for a time t=π/Ω. For the system in either |2〉+|3〉 or |1〉+|3〉 at time $t_{2}$, the probability for Alice obtaining a result |3〉 is zero, which leads to the paradox.

A condition similar to (64) gives an experimental criterion. The experiment of Kirchmair et al. [18] realises the nonlinear dynamics $H_{NL}$ for k=2 and generates cat states for α∼2.

Bob’s projective measurements can be performed by measuring the quadrature phase amplitude, along a chosen direction. For example, whether the system is in |3〉 or |4〉 can be determined by measuring $\hat{X}$. Similarly, whether the system is in |2〉 or |4〉 (or |1〉 or |4〉) can be determined by measuring along for the right choice of rotated axis. Consider the axes $\hat{X}$ and $\hat{P}$ rotated by an angle π/4 anti-clockwise about the origin. Denoting the rotated axes by ${\hat{X}}_{R}$ and ${\hat{P}}_{R}$, we see that whether the outcome of ${\hat{X}}_{R}$ is positive or negative distinguishes between the pair of states |2〉, |3〉 and the pair |1〉, |4〉. Bob opening the boxes |1〉 and 4〉 proceeds by a measurement of ${\hat{X}}_{R}$. If the sign of the outcome is positive, he knows the boxes 1 and 4 are empty, and no further measurement is performed. If the outcome is negative, he knows the ball is in box 1 or 4. He can then perform a measurement of ${\hat{P}}_{R}$ to determine which box, 1 or 4, the ball is in. The method of opening boxes 2 and 4 proceeds similarly. Bob measures the sign of ${\hat{P}}_{R}$ and if negative, the ball will be found in one of the boxes 2 or 4.

## 5. Finding Consistency with Weak Macroscopic Realism

### 5.1. The Weak Macroscopic Realism (wMR) Model

**Figure 8:**
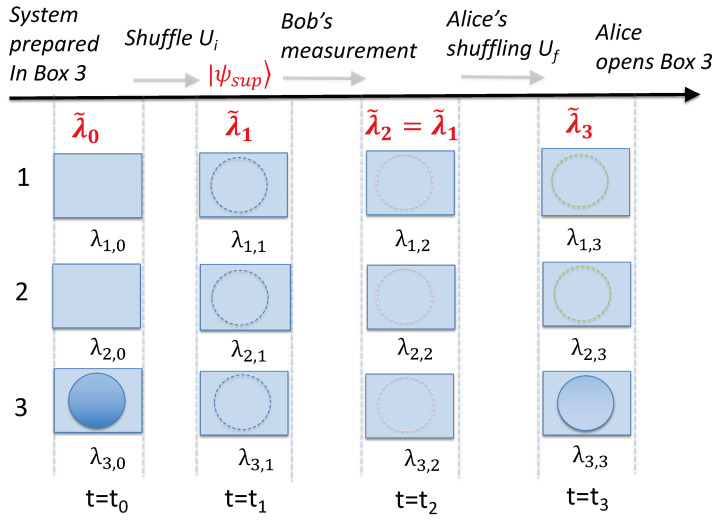
Schematic depicting the assumptions of the weak macroscopic realism (wMR) model for the three-box paradox. The ball is placed in box 3 at time $t_{0}$. After some unitary transformations, the system at time $t_{1}$ is in a superposition $|\psi_{sup}\rangle$ of being in one of the three boxes. Bob’s measurement of the system at time $t_{1}$ consists of opening either box 1 or box 2. Alice performs the unitary transformations $U_{f}$ on the system at time $t_{2}$, after Bob’s measurement, and opens box 3 at time $t_{3}$. According to wMR, at each time $t_{k}$, the outcome of finding the ball in a given box *I* or not is fixed, prior to the observer opening the box *I*. The predetermined outcome can be denoted by the variable $\lambda_{I,k}$. Also, wMR implies the ball will be found in only one box, if all boxes were to be opened. The predetermined outcome for which box the ball would be found in at time $t_{k}$ can be denoted by the variable ${\tilde{\lambda }}_{k}$. Bob’s measurement is assumed accurate, and his outcome will be determined by either $\lambda_{1,1}$ or $\lambda_{2,1}$. The values of $\lambda_{I,k}$ and ${\tilde{\lambda }}_{k}$ (but not the *details* about the states) are unchanged on measurement.

By definition, macroscopic realism (MR) posits that a system that has two or more macroscopically distinct states available to it will always be in one of those states [25]. According to MR, the ball at the times $t_{0}$, $t_{1}$, $t_{2}$, and $t_{3}$ is always in one of the boxes since the states in which the ball is found are macroscopically distinct. The relevant times $t_{k}$ (k=0,1,2,3) correspond to just after the initial preparation in state |3〉, just after the preparation of $|\psi_{1}\rangle =|\psi_{sup}\rangle$, just after Bob’s measurement (if it takes place), and after Alice’s final shuffling $U_{f}$ (Figure 8).

The meaning of “state” in the above definition is non-specific. Hence, there can be several different meanings of MR. In this paper, we consider that MR is defined in a minimal less-restrictive sense as *weak macroscopic realism* (wMR). We follow Refs. [20,21,22,23,28] and define wMR as follows. The definition in the context of the three-box paradox consists of two premises.

(1) A real property for the pointer measurement:

The premise of wMR specifies that the system is in some sort of state at time $t_{k}$, such that *the outcome for observing the ball in a given box or not is predetermined* at time $t_{k}$. For a given box *I*, this allows us to assign the variable $\lambda_{I,k}$ to the system defined at this time, to specify the outcome of such an observation—the ball being in the box I or not. It is also assumed as part of the definition of wMR that the ball can be in only one of the boxes at time $t_{k}$. Hence, we can specify the set of values $\left\{\lambda_{I,k}\right\}$ by one variable, ${\tilde{\lambda }}_{k}$, with this variable indicating which box the ball is in at time $t_{k}$.

In the context of the three-box experiment, we refer to the opening of the box by Alice or Bob as a “pointer measurement”. This is because the measurement occurs after the unitary operations corresponding to the shuffling and is likened to the reading of a final outcome in a Bell experiment, where one detects a photon or an electron at a given location, after the unitary operations corresponding to the passage through a Stern–Gerlach apparatus [47]. The predetermination proposed for the outcome of the measurement does not conflict with the results of Bell [47,48] or contextuality theorems [49] because the predetermination is defined for the system at time $t_{k}$, prepared with respect to the measurement basis. Other definitions of macroscopic realism exist where the unitary operations associated with the preparation of the measurement setting are not considered. These definitions of MR can be negated by Bell theorems that apply to macroscopic systems [20,25].

In a wMR model, the “state” of the system is not necessarily the same state as that of the system after the ball is observed, or after the preparation of the ball in a given box. In quantum mechanics, such a preparable state is an eigenstate of the measurement observable. Such issues have been carefully considered by Maroney [9] and Maroney and Timpson [50], who defined classes of macroscopic-realism models (although we point out that the authors use the term “macrorealism per se” to refer to what we call macroscopic realism (MR)). In the wMR model that we consider here (assuming the measurement is an effective one), the value of $\lambda_{I,k}$ is unchanged before and after the measurement in which an observer opens a box, even though the full description of the “state” itself may have changed (Figure 8). In a wMR model, there are no extra assumptions made about the details of the “state” of the ball—it cannot be assumed that the “state” is restricted to be a quantum state, for example [28]. It is not excluded that the underlying states might be fully ontological [51], or with an epistemic restriction at a microscopic level [52]. Compared with the classes developed by Maroney [9], models consistent with wMR would be included in the class of *supra eigenstate support macrorealism models* for macroscopic realism [9].

**Figure 9:**
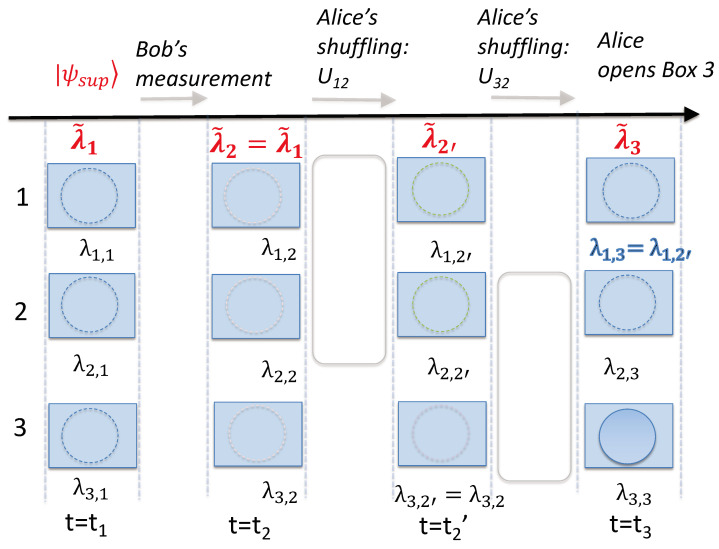
Schematic depicting the assumptions of the weak macroscopic realism (wMR) model. The model is as for Figure 8. Here, Alice’s transformation occurs in two stages. The first unitary acts only on boxes 1 and 2. According to wMR, premise (2), the value for $\lambda_{3,2}$ cannot change due to this operation (although nothing is inferred about the state of box 3 changing). Similarly, the second stage of Alice’s operation involving only boxes 2 and 3 cannot change the value of $\lambda_{1,2^{\prime }}$.

(2) A weak form of locality:

We also specify that *weak macroscopic realism* for each mode (box) *I* includes the assumption that the value $\lambda_{I,k}$ defined for the system at the time $t_{k}$ is not affected by any operations that might then occur *solely* at the other modes (Figure 9). This gives a meaning of a weak form of locality in the context of the three-box paradox. This form of locality is a subset of the conditions placed on a system by locality as defined by Bell [47].

*Comment:* Weak macroscopic realism differs from the assumption of macro-realism, which makes an additional assumption about the existence of non-invasive measurements. A full comparison is given in Section 6.

### 5.2. Finding Consistency with wMR

If it is assumed that *weak macroscopic realism* holds, then how does the three-box paradox, which seems to imply that the ball was simultaneously in two boxes, occur? We will address this question by analyzing the dynamics of the measurements, which include the unitary operations.

#### 5.2.1. Three-Box Mesoscopic Paradox

We first consider the standard three-box paradox and its mesoscopic realization, as in Section 3, where the three modes represent the three boxes. We demonstrate consistency with respect to both premises, wMR (1) and wMR (2).

*Premise wMR(1):* At the times $t_{1}$, $t_{2}$, and $t_{3}$, *after* the unitary transformations corresponding to the preparation or shuffling, wMR posits the system to have predetermined values for the final measurement of photon number $\hat{n}$ at each mode. By definition, wMR posits that the system in a macroscopic superposition state $|\psi_{k}\rangle$ at time $t_{k}$ can be described by a set of values $\lambda_{I,k}$ that determine the outcome of ${\hat{n}}_{I}$ for each mode [20]: the variable $\lambda_{I,k}$ assumes the value +1 or −1; here, 1 indicates the outcome to be *N*, and −1 indicates the outcome to be 0. We may also define the real property $\lambda_{k}$ as the state the system is in at the time $t_{k}$ i.e., whether the system will be identified by state |1〉, |2〉, or |3〉 after the observer opens the boxes.

**Figure 10:**
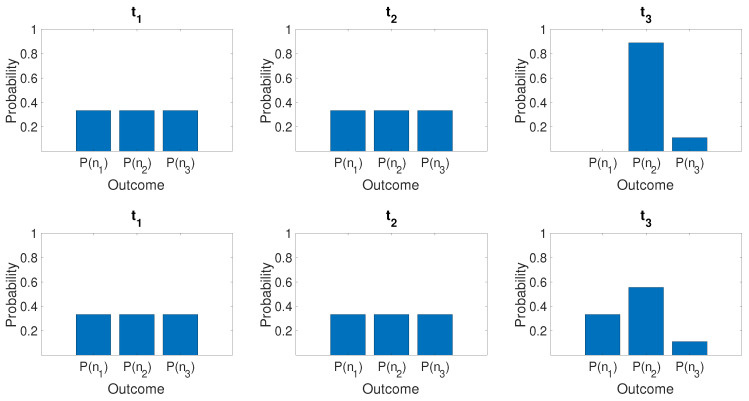
Measurable macroscopic differences occur for the probabilities of outcomes at time $t_{3}$, depending on whether Bob makes a measurement or not, but the differences emerge only *after* the dynamics of Alice’s operations (shuffling). This is consistent with weak macroscopic realism (wMR) since in the wMR model, the values $\lambda_{I,1}$ are assumed not to change with Bob’s measurement (Figure 8). The probabilities $P(n_{k})$ of obtaining *N* on measuring the photon number $\hat{n}$ of mode *k* at time *t* are plotted. The top sequence shows the sequence of probabilities if Bob makes no measurement. The lower sequence shows the probabilities if Bob makes a measurement between times $t_{1}$ and $t_{2}$. The plots on the right show the probabilities at time $t_{3}$, after Alice’s operation $U_{f}$, which takes place between times $t_{2}$ and $t_{3}$.

We argue that the existence of the real property $\lambda_{k}$ (or $\lambda_{I,k}$) is not inconsistent with the paradox. This is because the predictions for the outcomes of the pointer measurement as given by the superposition

(73) $$|\psi_{k}\rangle =\sum_{I=1,2,3}c_{I}|I\rangle$$

defined at time $t_{k}$ (where $c_{I}$ are probability amplitudes) are indistinguishable from those of a *mixture*

(74) $$\rho_{k}=\sum_{I=1,2,3}|c_{I}{|}^{2}\left|I\rangle \langle I\right|$$

for which there *is* a predetermination of the outcome of $\hat{n}$. The mixed state (74) models the system that will be *in* one of the states |1〉, |2〉, or |3〉, with probabilities $|c_{1}{|}^{2}$, $|c_{2}{|}^{2}$, and $|c_{3}{|}^{2}$, respectively. However, the system in $|\psi_{k}\rangle$ is not at time $t_{k}$ in any of the quantum eigenstates given by |1〉, |2〉, or |3〉. After the time $t_{k}$, unitary dynamics occurs, which leads to different final states for the superposition and the mixed state. This is illustrated in Figure 10, where we compare the dynamics if Bob does or does not make a measurement at time $t_{2}$. After his measurement, the system would collapse into the mixed state. The probabilities for the outcome of $\hat{n}$ for each mode immediately before and after Bob’s measurements are indistinguishable. However, *after* Alice’s unitary transformations, macroscopic differences occur.

*Premise wMR (2):* We now examine for consistency with the assumption of wMR that for each mode (box) *I*, the value $\lambda_{I,k}$ is not affected by any operations occurring solely at the other modes. Consider the case where Alice acts on the system in the state

(75) $$|\psi_{sup}\rangle =\frac{e^{i\varphi }}{\sqrt{3}}(|3\rangle +e^{i\varphi_{1}}(i|2\rangle -|1\rangle )).$$

We consider where the unitary parts $U_{21}$ and $U_{32}$ of Alice’s measurements are performed in sequence. Her unitary operation is

(76) $$U_{f}=U_{1f}^{-1}U_{2f}^{-1}$$

given by Equation (34). Alice performs $U_{21}\equiv U_{2f}^{-1}$ first, which involves only systems 1 and 2, arising from $H_{21}$. Then, wMR (2) posits that the hidden variable $\lambda_{3,2}$ defined for mode 3 at time $t_{2}$ after the single transformation $U_{21}$ *should not be affected by this transformation*.

**Figure 11:**
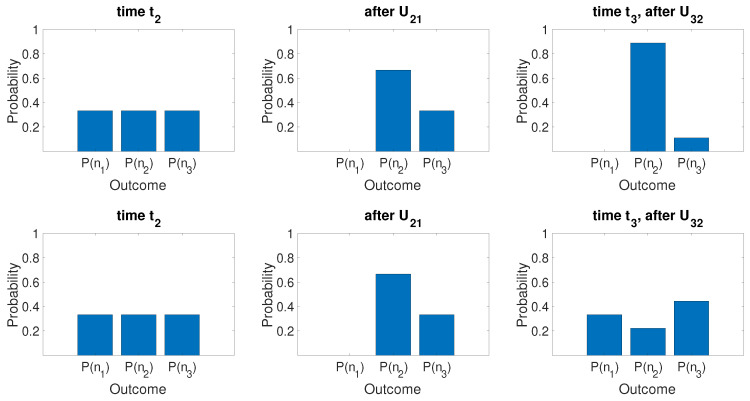
Validating the predictions of weak macroscopic realism (wMR): the sequence as Alice makes the operations $U_{f}$ on the states (**top**) $|\psi_{sup}\rangle =|\psi_{1}\rangle$ and (**lower**) $\rho_{3,mix}$, which is the state after Bob’s measurement detects the system in state |3〉 at time $t_{2}$. Alice first transforms (shuffling between boxes 1 and 2) according to $U_{f2}^{-1}=U_{21}$, leaving box 3 untouched. Then, she transforms (shuffling between boxes 3 and 2) by $U_{1f}^{-1}=U_{32}$ (see Figure 9). Far left are the plots of $P(n_{k})$ for the initial states (top) $|\psi_{sup}\rangle$ and (lower) $\rho_{mix}$. Second from left are the states after Alice’s transformation $U_{21}$. The predictions for $|\psi_{sup}\rangle$ and the mixture $\rho_{3,mix}$ are identical. Far right are the states after Alice’s transformation (shuffling between boxes 2 and 3) $U_{32}$, where the predictions diverge. The results are in agreement with the predictions of wMR (2), which posits that the system has a definite property $\lambda_{3,2}$ for box 3 (the ball is in the box or not) at time $t_{2}$, and this property cannot be changed by any shuffling $U_{21}$ on boxes 2 and 1. This is because throughout the shuffling $U_{21}$, the predictions are identical to those of the mixture $\rho_{3,mix}$, consistent with the definite value $\lambda_{3,2}$.

We now confirm that the predictions of the quantum paradox are indeed consistent with this hypothesis (Figure 11). We see that $U_{2f}=U_{2i}$. Hence, the state after this transformation is

(77) $$\begin{array}{ccc} U_{2f}^{-1}|\psi_{sup}\rangle & = & \frac{1}{\sqrt{2}}\left(\begin{array}{ccc} ie^{-i\varphi_{1}} & e^{-i\varphi_{1}} & 0\\ -ie^{-i\varphi_{1}} & e^{-i\varphi_{1}} & 0\\ 0 & 0 & \sqrt{2} \end{array}\right)\frac{e^{-i\varphi }}{\sqrt{3}}\left(\begin{array}{c} -e^{i\varphi_{1}}\\ ie^{i\varphi_{1}}\\ 1 \end{array}\right)\\ & = & \frac{e^{-i\varphi }}{\sqrt{6}}\left(\begin{array}{c} 0\\ 2i\\ \sqrt{2} \end{array}\right) \end{array}$$

which gives the probabilities 0, 2/3, and 1/3 for the detection of the system in states |1〉, |2〉, and |3〉. We next compare the evolution under $U_{21}\equiv U_{2f}^{-1}$ for the mixed state

(78) $$\begin{array}{ccc} \rho_{3,mix} & = & \frac{1}{3}(ie^{i\varphi_{1}}|2\rangle -e^{i\varphi_{1}}|1\rangle )(-ie^{-i\varphi_{1}}\langle 2|-e^{-i\varphi_{1}}\langle 1|)\\ & & +\frac{1}{3}|3\rangle \langle 3|. \end{array}$$

The system in (78) can be viewed as having a predetermined outcome for $\hat{n}$ on mode 3. Hence, there is a hidden variable $\lambda_{3,2}$ for the system in this description. Beginning with $\rho_{3,mix}$, the state after the transformation $U_{21}\equiv U_{2f}^{-1}$ is

(79) $$\begin{array}{ccc} \rho_{3,mix,21} & = & \frac{2}{3}\left|2\rangle \langle 2\right|+\frac{1}{3}\left|3\rangle \langle 3\right| \end{array}$$

which gives the same probabilities, 0, 2/3, and 1/3, as for $|\psi_{sup}\rangle$. There is consistency with wMR.

On the other hand, if we continue to evolve with $U_{32}\equiv U_{1f}^{-1}$, then the evolution of the two states $|\psi_{sup}\rangle$ and $\rho_{3,mix}$ diverges macroscopically. For $|\psi_{sup}\rangle$, we find

(80) $$\begin{array}{ccc} U_{1f}^{†}U_{2f}^{†}|\psi_{sup}\rangle & = & \frac{1}{\sqrt{3}}\left(\begin{array}{ccc} \sqrt{3} & 0 & 0\\ 0 & ie^{-i\varphi } & -e^{-i\varphi }\sqrt{2}\\ 0 & -ie^{-i\varphi }\sqrt{2} & -e^{-i\varphi } \end{array}\right)\\ & & \times \frac{e^{-i\varphi }}{\sqrt{6}}\left(\begin{array}{c} 0\\ 2i\\ \sqrt{2} \end{array}\right)\\ & = & \frac{e^{-i\varphi }}{\sqrt{18}}\left(\begin{array}{c} 0\\ -4e^{-i\varphi }\\ \sqrt{2}e^{-i\varphi } \end{array}\right). \end{array}$$

There is zero probability of finding the system in state |1〉. The relative probabilities are 0, 8/9, and 1/9. On the other hand, for the system initially in $\rho_{3,mix}$, the final state after both the transformations $U_{21}=U_{2f}^{-1}$ and then $U_{32}=U_{1f}^{-1}$ is

(81) $$\rho_{3,mix,F}=\frac{2}{3}U_{1f}^{-1}\left|2\rangle \langle 2\right|U_{1f}+\frac{1}{3}U_{1f}^{-1}\left|3\rangle \langle 3\right|U_{1f}$$

where

(82) $$U_{1f}^{-1}|2\rangle =\frac{e^{-i\varphi }}{\sqrt{3}}\left(\begin{array}{c} 0\\ i\\ -i\sqrt{2} \end{array}\right)$$

and

(83) $$U_{1f}^{-1}|1\rangle =\left(\begin{array}{c} 1\\ 0\\ 0 \end{array}\right).$$

The probability of finding the system in state |1〉 is 1/3. The evolution of $|\psi_{sup}\rangle$ leads to the paradox but does not contradict the premise of weak macroscopic realism because the local unitary transformation $U_{32}$ acts on both mode 3 and 2. The transformations are depicted in Figure 11.

#### 5.2.2. Cat-State Paradox

**Figure 12:**
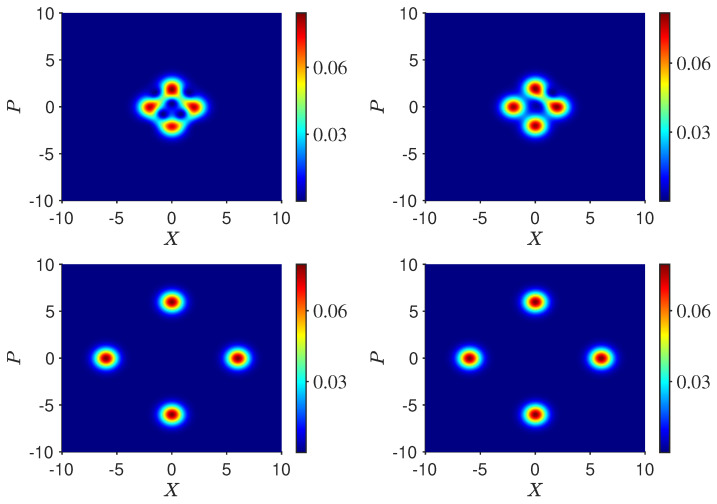
*Q* functions for the superposition $|\psi_{sup}\rangle$(Equation (44)) (**left**), and the mixture $\rho_{mix,14}$(Equation (86)) (**right**) become increasingly indistinguishable as the system size $\alpha_{0}$ increases. The top and lower pairs are for $\alpha_{0}=2$ and $\alpha_{0}=6$, respectively.

We now analyze the dynamics of the three-box paradox for the macroscopic example using coherent states, in order to show consistency with wMR. The paradox can be analyzed using the *Q* function, defined by [53]

(84) $$Q\left(\alpha \right)=\frac{\langle \alpha |\rho |\alpha \rangle }{\pi }$$

where ρ is the system density operator. We examine the case of k>2. The case of k=2 will show similar behavior. The system at time $t_{1}$ is in the superposition $|\psi_{sup}\rangle$ (Equation (44)). The Q function is

(85) $$\begin{array}{ccc} Q(x,p) & = & e^{-\frac{\left(|\alpha {|}^{2}+|\alpha_{0}{|}^{2}\right)}{2\pi }}(2\cos\left(x\alpha_{0}-p\alpha_{0}\right)\sinh\left(x\alpha_{0}-p\alpha_{0}\right)\\ & & -2\cos\left(x\alpha_{0}+p\alpha_{0}\right)\sinh\left(x\alpha_{0}+p\alpha_{0}\right)\\ & & +\cosh\left(2x\alpha_{0}\right)+\cosh\left(2p\alpha_{0}\right)\\ & & +\cos\left(2p\alpha_{0}\right)-\cos\left(2x\alpha_{0}\right)) \end{array}$$

where α=x+ip and $\alpha_{0}$ are taken to be real. The function is depicted by the contour graphs, in Figure 12.

Suppose Bob looks at boxes 1 and 4. After the projective measurement by Bob to determine whether the system is in state |1〉 or |4〉, or not (i=1,2), the system is in the mixed state given by the density operator

(86) $$\rho_{mix,14}=\frac{1}{4}\left|1\rangle \langle 1\right|+\frac{1}{4}\left|4\rangle \langle 4\right|+\frac{1}{4}(-|2\rangle +|3\rangle )(-\langle 2|+\langle 3|).$$

The Q function of the mixed state created at time $t_{2}$ is

(87) $$\begin{array}{ccc} Q_{mix,14}\left(x,p\right) & = & e^{-\frac{\left(|\alpha {|}^{2}+|\alpha_{0}{|}^{2}\right)}{2\pi }}(\cosh\left(2x\alpha_{0}\right)+\cosh\left(2p\alpha_{0}\right)\\ & & -\exp\left(x\alpha_{0}+p\alpha_{0}\right)\cos\left(x\alpha_{0}+p\alpha_{0}\right)) \end{array}$$

plotted in Figure 12. A comparison of Q(x,p) with $Q{\left(x,p\right)}_{mix,14}$ shows that as $\alpha_{0}\to \infty$, $Q\left(x,p\right)\to Q_{mix,14}\left(x,p\right)$. The terms contributing from the superposition are exponentially damped as $\alpha_{0}$ increases.

**Figure 13:**
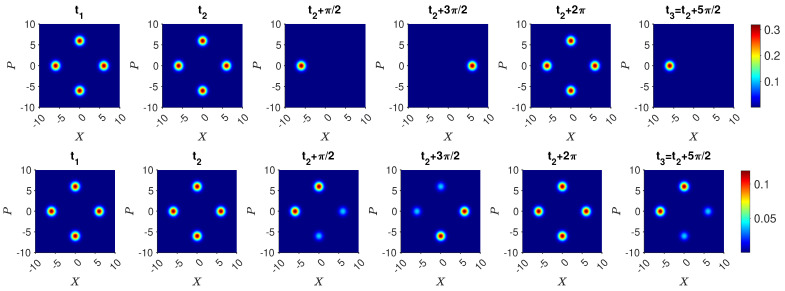
The dynamics of the macroscopic three-box paradox. The dynamics induced by Alice’s transformations $U_{f}$ are macroscopically sensitive to whether or not Bob has made a prior measurement of the system at time $t_{1}$, despite Bob’s measurement being seemingly non-invasive. The figure shows contour plots of Q(α) for the system prepared at time $t_{1}$ in thesuperposition state $|\psi_{sup}\rangle$ (Equation (44)) (**far left**) as it evolves under the action of Bob and Alice’s operations. The top sequence shows the dynamics ifthere is no measurement made by Bob. Alice makes her transformation $U_{f}$ on the system starting at time $t_{2}$ (**second from left**). The dynamics of the transformation $U_{f}$ are completed at time $t_{3}$. The lower sequence shows the evolution if Bob makes a measurement of the system at time $t_{1}$ (the outcome revealing whether the system is in the state |1〉 or |4〉, or not). At time $t_{2}$, after Bob’s measurement, the system is in the mixed state $\rho_{mix,14}$. While the *Q* function for $\rho_{mix,4}$ (**lower second from left**) is indistinguishable from that of $|\psi_{sup}\rangle$ (**top second from left**) as α→∞, indicating a non-invasive measurement, there is a macroscopic difference between the final probabilities (**top far right** and **lower far right**) after the dynamics of Alice’s transformations, at time $t_{3}$. Here, $\alpha_{0}=6$ and k=3.

Now, Alice performs the transformations. The system evolves according to $U_{f}$. The final state if Bob made no measurement is given by Equation (65) since the system remains in the superposition $|\psi_{sup}\rangle$ (Figure 13). On the other hand, the result if Bob opened boxes 1 and 4 is different. The system is $\rho_{mix,14}$ at time $t_{2}$. After the transformations $U_{f}$, the system is in the mixed state

(88) $$\begin{array}{ccc} \rho_{mix,14}\left(t_{3}\right) & = & \frac{1}{4}[U_{f}|1\rangle \langle 1|U_{f}^{†}]+\frac{1}{4}[U_{f}|4\rangle \langle 4|U_{f}^{†}]\\ & & +\frac{1}{2}U_{f}\left(\frac{-|2\rangle +|3\rangle }{\sqrt{2}}\right)\left(\frac{-\langle 2|+\langle 3|}{\sqrt{2}}\right)U_{f}^{†}. \end{array}$$

The states $U_{f}|1\rangle$, $U_{f}|4\rangle$, and $U_{f}(-|2\rangle +\left|3\rangle \right)/\sqrt{2}$ are given by Equations (55), (56) and (58). Figure 13 shows the final state after Alice’s operations $U_{f}$, for the two initial states $|\psi_{sup}\rangle$ and $\rho_{mix,14}$ at time $t_{2}$. While the *Q* functions are initially indistinguishable, after $U_{f}$, a macroscopic difference between the final states emerges. This is consistent with a model in which weak macroscopic realism holds but where there is measurement disturbance due to Bob’s interactions.

#### 5.2.3. Summary

On examining the dynamics $U_{f}$, the paradox is seen to arise from the distinction between the state before and after Bob’s measurement, a distinction that is initially microscopic in the sense that it is not detectable by any difference between observable probabilities for whether the ball is in a given box, or, as in Figure 13, by an observable difference between *Q* functions. In fact, as for Figure 12, the overlap between the states before and after Bob’s measurement approaches unity as the system size increases. This gives the seemingly paradoxical situation whereby the measurement disturbance from Bob vanishes, but the effect of it nonetheless can be extracted by suitable dynamics.

## 6. Leggett–Garg Test of Macro-Realism

The Leggett–Garg inequality can be violated for systems that do not jointly satisfy the combined premise of macroscopic realism and no measurement-disturbance. Leggett and Garg considered a system that at certain times can be found to be in one of two macroscopically distinguishable states. A definition of macroscopic realism (MR) was proposed by Leggett and Garg [25]: macroscopic realism asserts that the system is at each time always in one or the other of the two macroscopically distinct states. Leggett and Garg considered a measurable quantity $Q_{k}$ that would distinguish the states at time $t_{k}$, taking on the values +1 and −1 for the respective outcomes. According to MR, a variable $\lambda_{k}$ can be ascribed to the system at time $t_{k}$, where we let $\lambda_{k}=+1$ or −1, depending on which macroscopic state the system is in. Where the system is in a superposition of macroscopically distinct states, this variable may be thought of as “hidden” since it is not part of the quantum description. If non-invasive measurability (NIM) holds, there is no disturbance to the future dynamics of the system from a non-invasive measurement made to determine the value of $\lambda_{k}$ (or $Q_{k}$) at time $t_{k}$. It is then possible to define two-time moments where $\langle Q_{i}Q_{j}\rangle =\langle \lambda_{i}\lambda_{j}\rangle$. In this case, *macro-realism* is said to hold, and the Leggett–Garg inequalities follow [25].

The application of a Leggett–Garg test to the three-box paradox elucidates the origin of the paradox [9]. Maroney derived new versions of the Leggett–Garg inequality that apply to the paradox. The Condition 1 of Ref. [9] is satisfied because

(89) $$P_{N,A}\left(3_{3}\right)=P_{B1,A}\left(3_{3}\right)=P_{B2,A}\left(3_{3}\right)$$

as explained in Section 2. This means that Alice observes no change in the probability of her detecting a ball in box 3 due to whether Bob opens one of the boxes or not. Maroney refers to Bob’s measurement, which satisfies this condition, as operationally non-disturbing. Maroney pointed out that while this condition is satisfied in the three-box paradox, it has not been satisfied in other tests of Leggett–Garg inequalities. Hence, this condition allows for a stronger test of macro-realism. Here, we illustrate that the mesoscopic paradox of Section 3 will satisfy the strict Maroney–Leggett–Garg test of macro-realism and that violation of a Leggett–Garg inequality is also possible for the macroscopic system of Section 4.

### 6.1. Weak Macroscopic Realism (wMR) and the Leggett–Garg Assumptions

In the analysis of the Leggett–Garg inequalities, wMR as defined in Section 5 is sufficient to allow for the specification of the variable $\lambda_{k}$ that predetermines the outcome of $Q_{k}$, as introduced by Leggett and Garg. According to wMR, the ball at the times $t_{0}$, $t_{1}$, $t_{2}$, and $t_{3}$ is always *in* one of the boxes, if these boxes correspond to macroscopically distinct states. The times correspond to just after the initial preparation of the ball in box 3, just after the creation of the superposition state $|\psi_{1}\rangle =|\psi_{sup}\rangle$, just after Bob’s measurement (if it takes place), and after Alice’s final shuffling. The meaning of the “ball being in a box” refers to the predetermination of the outcome if the observer looks in the box and is not intended to fully specify details at a microscopic level about the state of the ball itself. The variable $\lambda_{I,k}$ ensures a predetermination of the outcome of Bob or Alice looking in any particular box *I*, at time $t_{k}$, k=0,1,2,3. Since we also assume that the ball can be in only one of the boxes, we can specify the set of values $\left\{\lambda_{I,k}\right\}$ by *one* variable, $\lambda_{k}$, which indicates which box the ball is in at time $t_{k}$. The wMR model does not include the assumption of NIM, and hence wMR is not negated by the violation of Leggett–Garg inequalities.

Maroney and Timpson introduced different models for macroscopic realism, as applied to a single system [9,50]. The wMR models can correspond to the class referred to by Maroney as “supra eigenstate support macrorealism”, in which the states of the system do not necessarily correspond to the states that are prepared when a ball is placed in a box [9]. Hence, wMR allows the state of the system to be changed on measurement, even when the value of $\lambda_{k}$ is not. This is consistent with the behavior illustrated for the *Q* function before and after Bob’s measurement, shown in Figure 13. In this paper, we have extended the definition to bipartite systems by considering the premise wMR (2) in Section 5. However, for the Leggett–Garg inequalities considered in this section, we do not invoke the premise wMR (2) but consider only the variable $\lambda_{k}$, which describes the entire system.

### 6.2. Original Three-Box Paradox and the Mesoscopic Realization

**Figure 14:**
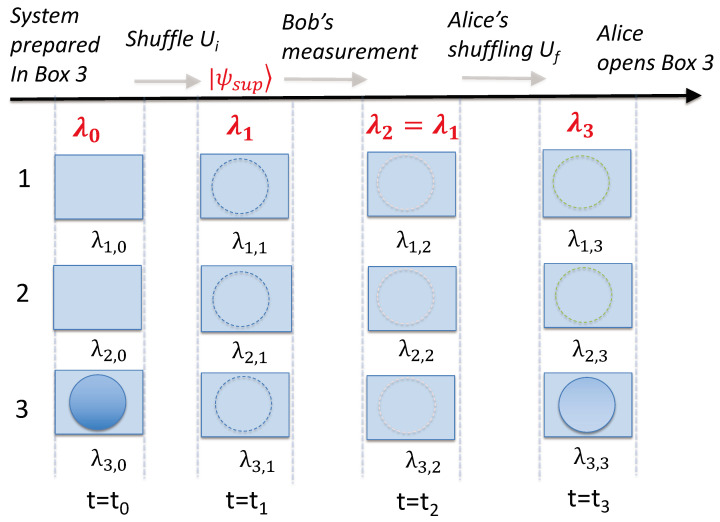
Schematic depicting how the weak macroscopic realism (wMR) model can be consistent with the strong Maroney–Leggett–Garg (MLG) test of macro-realism. The notation is as for Figure 8. In the proposed MLG test, the variable $\lambda_{k}$ takes the value −1 if the ball is in box 1 or 2, and +1 if the ball is in box 3. The MLG test gives justification of non-invasive measurability (NIM) because it can be verified that $\langle \lambda_{0}\lambda_{3}\rangle$ is unchanged depending on whether Bob makes a measurement or not (Condition (89)). Bob cannot fully evaluate $\lambda_{1}$ by his measurement in which he opens either box 1 or 2. His measurement does not change the value of $\lambda_{1}$, but it may, however, change the state of the system. This implies that the unitary operations $U_{f}$ employed by Alice can result in a different state of the system at time $t_{3}$, and hence different correlations $\langle \lambda_{1}\lambda_{3}\rangle$, depending on whether Bob makes a measurement.

We first consider the original three-box paradox and its mesoscopic version as in Section 3. Following Maroney [9], we distinguish between the two distinct states defined by the ball being in box 1 or 2, or else the ball being in box 3 (Figure 14). The premise of wMR implies that the result of the observation of the ball being in any given box or not is predetermined. Accordingly, we define the variable to be $\lambda_{k}=-1$ if the result would indicate the ball is in box 1 or 2 (i.e., states |1〉 or |2〉), and $\lambda_{k}=1$ if the result would indicate the ball is in box 3 (state |3〉). In the mesoscopic version, these states are mesoscopically distinct, but where *N* is large, the states become macroscopically distinct.

The times considered are $t_{0}$, $t_{1}$, and $t_{3}$. It is readily shown, by considering all possible values for $\lambda_{k}$ at each time, that algebraically, $-1\leq \lambda_{0}\lambda_{1}+\lambda_{1}\lambda_{3}+\lambda_{0}\lambda_{3}\leq 3$ [25]. At time $t_{0}$, the ball is always prepared in box 3, and $\lambda_{0}=1$. Assuming NIM, the values of each $\lambda_{k}$ can be measured non-invasively. Hence, the premises of macro-realism imply the Leggett–Garg inequality $-1\leq Q_{LG}\leq 3$, where

(90) $$\begin{array}{ccc} Q_{LG} & = & \langle Q_{0}Q_{1}\rangle +\langle Q_{1}Q_{3}\rangle +\langle Q_{0}Q_{3}\rangle \\ & \equiv & \langle \lambda_{0}\lambda_{1}\rangle +\langle \lambda_{1}\lambda_{3}\rangle +\langle \lambda_{0}\lambda_{3}\rangle . \end{array}$$

In fact, the measurement of $\lambda_{0}$ is not necessary because its value is known on preparation. The final measurement at time $t_{3}$ is assumed to be an accurate measurement of the state of the system. The measurement of the system at time $t_{1}$, however, is assumed to be non-invasive. Methods to justify this are discussed in Refs. [9,25] and include the use of an ideal negative result measurement, in which the ball is only ever determined to be absent in a box.

We now summarize the work of Maroney since this will apply directly to the mesoscopic example. The link with the condition for a three-box paradox can be made since the premise of wMR leads to constraints on the relations between the probabilities. Using the initial condition, we find that

(91) $$Q_{LG}=\langle \lambda_{1}\rangle +\langle \lambda_{3}\rangle +\langle \lambda_{1}\lambda_{3}\rangle .$$

Consistent with the wMR model, we define within the wMR model the probability that the system is in box I at time $t_{k}$ as $P(I_{k}$). In this model, the ball must be in one of the boxes: $\sum_{I=1,2,3}P\left(I_{k}\right)=1$. Considering the system prepared at time $t_{1}$, Bob can make measurements to reveal the state of the system. These measurements take place at time $t_{2}$. Strictly, time $t_{2}$ is defined to be the time after Bob’s measurements. However, since we assume the measurement by Bob in opening a box will accurately reveal the value of $\lambda_{I,k}$, we can say that $P\left(I_{1}\right)=P\left(I_{2}\right)$. Hence, wMR implies

(92) $$\begin{array}{ccc} \langle \lambda_{1}\rangle & = & -P\left(1_{1}\right)-P\left(2_{1}\right)+P\left(3_{1}\right)\\ & = & -P\left(1_{2}\right)-P\left(2_{2}\right)+P\left(3_{2}\right)\\ & = & 2P(3_{2})-1 \end{array}$$

Similarly,

(93) $$\langle \lambda_{3}\rangle =2P\left(3_{3}\right)-1$$

We note that the value $\langle \lambda_{3}\rangle$ can be evaluated in two ways. The first is where Bob makes no measurement of the system, so that the system at time $t_{2}$ is unchanged from that at time $t_{1}$, and the second is where Bob makes his measurement. Since it is assumed that it is possible to make a non-invasive measurement, the standard approach is to evaluate $\langle \lambda_{3}\rangle$ by the first method. In this example, $P_{B1,A}\left(3_{3}\right)=P_{N,A}\left(3_{3}\right)$, and the two approaches give the same value. In the present example, we note that the value of $\lambda_{2}$ is not directly measured by Bob’s measurement.

The wMR model posits that the ball is predetermined to be found in only one of the boxes at each time $t_{k}$, implying

(94) $$P\left(3_{2}\right)=P\left(3_{2},1_{3}\right)+P\left(3_{2},2_{3}\right)+P\left(3_{2},3_{3}\right)$$

and similarly,

(95) $$P\left(3_{3}\right)=P\left(1_{2},3_{3}\right)+P\left(2_{2},3_{3}\right)+P\left(3_{2},3_{3}\right).$$

Here, the joint probabilities that the ball is to be found in boxes I and J at times *K* and *L*, respectively, are denoted $P(I_{K},J_{L}$). The wMR premise also implies relations between joint probabilities and marginals, for example,

(96) $$\begin{array}{ccc} P(1_{2}+2_{2},1_{3}+2_{3}+3_{3}) & = & \sum_{I_{2}=1,2}\sum_{J_{3}=1,2,3}P\left(I_{2},J_{3}\right)\\ & = & P(1_{2}+2_{2})\\ & = & 1-P(3_{2}) \end{array}$$

where $P(1_{2}+2_{2})$ denotes the probability for the system being in boxes 1 or 2 at time $t_{2}$. Hence, wMR implies

(97) $$\begin{array}{ccc} \langle \lambda_{1}\lambda_{3}\rangle & = & P\left(3_{2},3_{3}\right)+P\left(2_{2},2_{3}\right)+P\left(1_{2},1_{3}\right)+P\left(1_{2},2_{3}\right)\\ & & +P\left(2_{2},1_{3}\right)-P\left(3_{2},1_{3}\right)-P\left(3_{2},2_{3}\right)\\ & & -P\left(1_{2},3_{3}\right)-P\left(2_{2},3_{3}\right)\\ & = & P\left(3_{2},3_{3}\right)+1-P\left(3_{2}\right)-\left(P\left(3_{3}\right)-P\left(3_{2},3_{3}\right)\right)\\ & & -\left(P\left(3_{3}\right)-P\left(3_{2},3_{3}\right)\right)-\left(P\left(3_{2}\right)-P\left(3_{2},3_{3}\right)\right). \end{array}$$

This leads to

(98) $$\begin{array}{ccc} Q_{LG} & = & 4P(3_{2},3_{3})-1\\ & = & 4(P\left(3_{3}\right)-P\left(1_{2},3_{3}\right)-P\left(2_{2},3_{3}\right))-1\\ & = & 4P\left(3_{3}\right)\left(1-P\left(1_{2}|3_{3}\right)-P\left(2_{2}|3_{3}\right)\right)-1 \end{array}$$

which becomes a measurable inequality with the assumption of NIM. Hence, the Leggett–Garg inequality

(99) $$Q_{LG}<-1$$

becomes

(100) $$P\left(1_{2}|3_{3}\right)+P\left(2_{2}|3_{3}\right)>1.$$

The condition presented in [9] beautifully encapsulates the nature of the three-box paradox. The $P_{B1,A}\left(1_{2},3_{3}\right)$ is the joint probability that the ball be found in box 1 at time $t_{1}$ and box 3 at time $t_{3}$, as measured over the subensemble when Bob opens box 1. Assuming NIM, Bob’s measurement and choice to open either box 1 or box 2 will not affect the statistics. With this assumption, condition (100) becomes

(101) $$P_{B1,A}\left(1_{2}|3_{3}\right)+P_{B2,A}\left(2_{2},3_{3}\right)>1$$

which is condition (17) for the observation of the three-box paradox.

Considering the paradoxes of Section 2 and Section 3, the probability of Alice detecting a ball in box 3 at time $t_{3}$, regardless of a measurement by Bob, is $P(3_{3})=1/9$. The joint probability $P_{B1,A}\left(1_{2},3_{3}\right)$ is measurable by Alice and Bob and calculable as $P\left(1_{2}\right)P\left(3_{3}|1_{2}\right)$. Here, $P(1_{2})=1/3$ and $P(3_{3}|1_{2})=1/3$, from the earlier sections. Alternatively, $P\left(1_{2},3_{3}\right)=P\left(3_{3}\right)P\left(1_{2}|3_{3}\right)=P\left(3_{3}\right)=1/9$ since $P(1_{2}|3_{3})=1$. The results for $P(2_{2},3_{3})$ are identical, giving $Q_{LG}=-13/9$, which is a violation of the Leggett–Garg inequality, implying a negation of macro-realism, as shown by Maroney [9]. The test can be performed using the mesoscopic version of Section 3 since the predictions are identical to the original paradox. Maroney emphasized the importance of the operational non-disturbing result (89) being used to justify NIM.

### 6.3. Macroscopic Cat-State Realization of “Three-Box” Paradox

The Leggett–Garg test is also applicable to the macroscopic set-up proposed in Section 4. At the times $t_{0}$, $t_{1}$, $t_{2}$, and $t_{3}$, the premise of weak macroscopic realism (wMR) posits that the system has a predetermined outcome for the measurement that distinguishes between the four coherent states, in the large α limit. As above, a variable can be defined to denote this predetermination. As above, we consider the three times, $t_{0}$, $t_{1}$, and $t_{3}$ as defining the Leggett–Garg test.

We let $\lambda_{k}=-1$ if the system at time $t_{k}$ is to be found in |1〉 or |4〉, or |2〉 or |4〉, and $\lambda_{k}=1$ if the system is to be found in |3〉. At time $t_{0}$, the ball is in |3〉, and $\lambda_{0}=1$. We find $\langle \lambda_{1}\rangle =2P\left(3_{2}\right)-1$ and $\langle \lambda_{3}\rangle =2P\left(3_{3}\right)-1$, as above.

There are different versions of a Leggett–Garg test that one can consider. The standard approach is to consider the statistics at the different times $t_{k}$, as though Bob can in principle make a non-invasive measurement at time $t_{2}$. However, this does not always allow for an operationally non-disturbing measurement [9]. The value $\langle \lambda_{3}\rangle$ can be evaluated in two ways, depending on whether Bob makes an intermediate measurement of the state at time $t_{1}$. In this example, the two methods will differ. This is also the case for standard tests of Leggett–Garg inequalities, where the evaluation of $\langle \lambda_{2}\rangle$ is selected to be without Bob’s measurement, on the basis that the ideal measurement would be non-invasive. (In fact, if the measurement of the system at time $t_{1}$ gives the value of $\lambda_{1}$, then no violation is possible when the intermediate measurement is made since all the values of $\lambda_{k}$ are known and the algebraic relations leading to the Leggett–Garg inequality are satisfied.)

In the present example, we propose to consider a modified Leggett–Garg test as follows. We denote by $P_{B1,B4}\left(3_{3}\right)$ the probability when Bob makes a measurement on the state at time $t_{1}$ by opening boxes 1 and 4, and Alice opens box 3 at the later time $t_{3}$. $P_{B2,B4,A}\left(3_{3}\right)$ is defined similarly, when Bob opens boxes 2 and 4. These probabilities are equal and will be denoted $P_{Bx,B4,A}\left(3_{3}\right)$. The value for $P(3_{3})$ when Bob makes no measurement at $t_{1}$ is denoted $P_{N,A}\left(3_{3}\right)$ and is different to $P_{Bx,B4,A}\left(3_{3}\right)$. This leads to two possible values of $Q_{LG}$: the first where $\langle \lambda_{3}\rangle$ is measured with a measurement at $t_{1}$, and the second when $\langle \lambda_{3}\rangle$ is measured without the measurement at $t_{1}$.

To avoid this problem, we will consider that the times $t_{k}$ are defined with the knowledge that Bob will make a measurement of the system defined at time $t_{1}$. The premise wMR will still apply to the system at each of the times $t_{k}$ (k=0,1,2,3), and the variables $\lambda_{k}$ can be defined. The measurement choice is that Bob either opens boxes 1 and 4, or boxes 2 and 4. It is no longer assumed that the measurement itself does not affect the value of $\lambda_{3}$, but rather the NIM premise is that Bob’s choice of measurement does not affect $\lambda_{3}$. Since this choice does not change the statistics for $P(3_{3})$, the NIM premise appears reasonable.

We now consider $Q_{LG}=\langle \lambda_{0}\lambda_{1}\rangle +\langle \lambda_{1}\lambda_{3}\rangle +\langle \lambda_{0}\lambda_{3}\rangle \equiv \langle \lambda_{1}\rangle +\langle \lambda_{1}\lambda_{3}\rangle +\langle \lambda_{3}\rangle$ according to the wMR model. To derive $\langle \lambda_{1}\lambda_{3}\rangle$, we extend the logic above to apply to the four possible states: here, we use that wMR posits the value of $\lambda_{I,2}=\lambda_{I,1}$, meaning that the outcome of Bob’s measurement reflects the state of the ball at time $t_{1}$. Hence,

(102) $$\begin{array}{ccc} P(3_{2}) & = & P\left(3_{2},1_{3}\right)+P\left(3_{2},2_{3}\right)+P\left(3_{2},3_{3}\right)+P\left(3_{2},4_{3}\right) \end{array}$$

and similarly

(103) $$\begin{array}{ccc} P(3_{3}) & = & P\left(1_{2},3_{3}\right)+P\left(2_{2},3_{3}\right)+P\left(3_{2},3_{3}\right)+P\left(4_{2},3_{3}\right). \end{array}$$

Also,

(104) $$\begin{array}{ccc} P(1_{2}+2_{2}+4_{2},1_{3}+2_{3}+4_{3}+3_{3}) & = & \sum_{I_{2}=1,2,4}\sum_{J_{3}}P\left(I_{2},J_{3}\right)\\ & = & P(1_{2}+2_{2}+4_{2})\\ & = & 1-P(3_{2}). \end{array}$$

We continue as above: first, we see that

(105) $$\begin{array}{ccc} \langle \lambda_{1}\lambda_{3}\rangle & = & P(3_{2},3_{3})+P\left(4_{2},4_{3}\right)+P\left(4_{2},1_{3}\right)+P\left(4_{2},2_{3}\right)+\\ & & +P\left(2_{2},1_{3}\right)+P\left(2_{2},2_{3}\right)+P\left(2_{2},4_{3}\right)+P\left(1_{2},1_{3}\right)\\ & & +P\left(1_{2},2_{3}\right)+P\left(1_{2},4_{3}\right)-P\left(3_{2},1_{3}\right)-P\left(3_{2},2_{3}\right)\\ & & -P(3_{2},4_{3})-P\left(1_{2},3_{3}\right)-P\left(2_{2},3_{3}\right)-P\left(4_{2},3_{3}\right). \end{array}$$

We can simplify to

(106) $$\begin{array}{ccc} \langle \lambda_{1}\lambda_{3}\rangle & = & P\left(3_{2},3_{3}\right)-\left(P\left(3_{2}\right)-P\left(3_{2},3_{3}\right)\right)\\ & & -\{P\left(1_{2}+2_{2}+4_{2},3_{3}\right)\\ & & -P(1_{2}+2_{2}+4_{2},1_{3}+2_{3}+4_{3})\}\\ & = & P\left(3_{2},3_{3}\right)-\left(P\left(3_{2}\right)-P\left(3_{2},3_{3}\right)\right)\\ & & -\{P\left(3_{3}\right)-P\left(3_{2},3_{3}\right)\}\\ & & +\{P\left(1_{2}+2_{2}+4_{2}\right)-P\left(1_{2}+2_{2}+4_{2},3_{3}\right)\}, \end{array}$$

and finally express in terms of the probabilities for box 3 only

(107) $$\begin{array}{ccc} \langle \lambda_{1}\lambda_{3}\rangle & = & P\left(3_{2},3_{3}\right)-\left(P\left(3_{2}\right)-P\left(3_{2},3_{3}\right)\right)\\ & & -\{P\left(3_{3}\right)-P\left(3_{2},3_{3}\right)\}\\ & & +1-P\left(3_{2}\right)-\left\{P\left(3_{3}\right)-P\left(3_{2},3_{3}\right)\right\}\\ & = & 4P\left(3_{2},3_{3}\right)+1-2P\left(3_{2}\right)-2P\left(3_{3}\right). \end{array}$$

We now consider $Q_{LG}=\langle \lambda_{1}\lambda_{3}\rangle +\langle \lambda_{1}\rangle +\langle \lambda_{3}\rangle$ according to the wMR model. The state at time $t_{3}$ is defined with the measurement of Bob in place. Now, $\langle \lambda_{1}\rangle =2P\left(3_{2}\right)-1$ from wMR. Hence,

(108) $$\begin{array}{ccc} \langle \lambda_{1}\lambda_{3}\rangle +\langle \lambda_{1}\rangle & = & 4P\left(3_{2},3_{3}\right)-2P\left(3_{3}\right). \end{array}$$

The NIM premise is that Bob’s choice of measurement does not affect $\lambda_{3}$. Since this choice does not change the statistics for $P(3_{3})$, the NIM premise appears reasonable. This allows us to posit that $P\left(3_{3}\right)\equiv P_{Bx,B4,A}\left(3_{3}\right)$, and hence

(109) $$\langle \lambda_{3}\rangle =2P_{Bx,B4,A}\left(3_{3}\right)-1.$$

We find

(110) $$\begin{array}{ccc} Q_{LG} & = & \langle \lambda_{1}\lambda_{3}\rangle +\langle \lambda_{1}\rangle +\langle \lambda_{3}\rangle \\ & = & 4P(3_{2},3_{3})-1. \end{array}$$

Now, we know that since the ball can only be in one box, the wMR model implies $P\left(\left\{1_{2},4_{2}\right\},3_{3}\right)=P\left(1_{2},3_{3}\right)+P\left(4_{2},3_{3}\right)$. Hence,

(111) $$\begin{array}{ccc} Q_{LG} & = & 4[P\left(3_{3}\right)-P\left(\left\{1_{2},4_{2}\right\},3_{3}\right)-P\left(\left\{2_{2},4_{2}\right\},3_{3}\right)\\ & & +P(4_{2},3_{3})-1]. \end{array}$$

We now use NIM premise to write

(112) $$\begin{array}{ccc} Q_{LG} & = & 4(P_{Bx,B4,A}\left(3_{3}\right)-P_{B1,B4,A}\left(\left\{1_{2},4_{2}\right\},3_{3}\right)\\ & & -P_{B2,B4,A}\left(\left\{2_{2},4_{2}\right\},3_{3}\right)+P_{Bx,B4,A}\left(4_{2},3_{3}\right))-1\\ & = & 4P_{Bx,B4,A}\left(3_{3}\right)\{1-P_{B1,B4,A}\left(\left\{1_{2},4_{2}\right\}|3_{3}\right)\\ & & -P_{B2,B4,A}\left(\left\{2_{2},4_{2}\right\}|3_{3}\right)+P_{Bx,B4,A}\left(4_{2}|3_{3}\right)\}-1 \end{array}$$

where we note again that $P_{B1,B4,A}\left(4_{2}|3_{3}\right)=P_{B2,B4,A}\left(4_{2}|3_{3}\right)$ and $P_{B1,B4,A}\left(3_{3}\right)=P_{B2,B4,A}\left(3_{3}\right)$. Hence, the violation of the Leggett–Garg inequality $Q_{LG}<-1$ will occur when

(113) $$\begin{array}{c} P_{B1,B4,A}\left(\left\{1_{2},4_{2}\right\}|3_{3}\right)+P_{B2,B4,A}\left(\left\{2_{2},4_{2}\right\}|3_{3}\right)\\ >1+P_{B1,B4,A}\left(4_{2}|3_{3}\right) \end{array}$$

which is precisely the experimental condition (64) given for the paradox. This result justifies the earlier conclusion for this condition. The condition is that required for the violation of the Leggett-Garg inequality. Using the solutions $P_{B1,B4}\left(3_{3}\right)=P_{B1,B2}\left(3_{3}\right)=1/8$, $P_{B1,B4}\left(4_{2}|3_{3}\right)=1/2$, $P_{B1,B4}\left(1_{2},4_{2}|3_{3}\right)=1$, and $P_{B2,B4}\left(2_{2},4_{2}|3_{3}\right)=1$, we find $Q_{LG}=-5/4$, which gives a violation of the Leggett–Garg inequality and hence a negation of macro-realism.

### 6.4. Finding Consistency with Weak Macroscopic Realism

We ask: What can be concluded from the violations of the Leggett–Garg inequalities? If we allow consistency with wMR, then we would infer that the premise of NIM fails.

The assumption of weak macroscopic realism posits the validity of the variables $\lambda_{k}$ and the resulting probabilistic relationships derived in this section, as in Ref. [9]. We note that wMR implies that the value of $\lambda_{k}$ is the outcome of the measurement at time $t_{k}$ but that the state of the system after the measurement can be different to that before. Quantum mechanically, the state after measurement is an eigenstate of the measurement observable and would (if measured immediately without any shuffling) hence give the same value $\lambda_{k}$ for a subsequent measurement. The measurement is noninvasive with respect to the macroscopic property given by $\lambda_{k}$.

However, in the wMR model, the state of the system changes with the measurement—the measurement is invasive, even if microscopically so. The difference leads to a macroscopic change over the time duration of the unitary dynamics associated with Alice’s measurements. The operational non-disturbing condition is a necessary condition to demonstrate the non-invasiveness of Bob’s measurement on the system defined at time $t_{1}$, but it is not a sufficient condition. The measurement is shown not to change the statistics for $\langle \lambda_{0}\lambda_{3}\rangle$, but it cannot be inferred that the correlations $\langle \lambda_{1}\lambda_{3}\rangle$ do not change.

## 7. Conclusions

This paper gives proposals for mesoscopic and macroscopic quantum three-box paradoxes. The unitary operations (shuffling) required for the three-box paradox are realized by nonlinear interactions, which we model by specific Hamiltonians. The motivation for considering the macroscopic versions is to argue the case for realism: generally, the paradox may be explained as a failure of realism or else explained by measurement disturbance.

We show how macroscopic realism can be upheld consistently with the paradox. Macroscopic realism asserts that the system with two macroscopically distinct states available to it has a predetermined value for the outcome of a measurement that distinguishes those states. In order to achieve consistency with macroscopic realism, the definition of macroscopic realism is refined so that it applies to the system created at time $t_{i}$ *after* the unitary operations that determine the local measurement basis. The definition includes that the predetermined value cannot then be changed (after $t_{i}$) by any operations or measurements on spatially separated systems. We refer to this restricted definition as *weak macroscopic realism* (wMR). Weak macroscopic realism has been shown to be consistent with violations of macroscopic Bell inequalities [20].

Following Maroney [9], we have demonstrated that the realization of the paradox corresponds to a violation of a Leggett–Garg inequality. Hence, the combined assumptions of macroscopic realism and noninvasive measurability (macro-realism) are negated by the paradox. Our proposals, however, are macroscopic and have the advantage that macro-realism is tested in the spirit of the Leggett–Garg paper [25], applying to a system where macroscopic realism can be genuinely applied. We illustrate how the Leggett–Garg inequality can be violated and yet macroscopic realism upheld, with the violation occurring due to a failure of noninvasive measurability.

Further, in this paper we illustrate the paradoxical features of the measurement disturbance by manipulating the parameter that determines the size of the system. The disturbance becomes minimal with increasing size, yet the probabilities after Alice’s unitary operations remain macroscopically distinguishable, depending on whether a measurement occurred or not. This effect is similar to a quantum revival and we expect that the origin is non-classical.

The definition of macroscopic realism is required to be minimal. Macroscopic realism posits that there is a predetermined value for the outcome of the macroscopic measurement: This means that the ball is either in the box or not prior to Alice or Bob opening the box. However, it can be shown that if the system is viewed as being in a ‘state’ with the predetermined outcome + or −, then that ‘state’ cannot be given as a quantum state $|\psi_{+}\rangle$ or $|\psi_{-}\rangle$, prior to measurement [20]. This points to an inconsistency between wMR and (the standard interpretation of) quantum mechanics, as in Schrödinger’s argument [24]. The acceptance of wMR as part of the explanation of the paradox may raise other open questions concerning the completeness of quantum mechanics.

Finally, we consider the possibility of an experiment. The unitary dynamics required for the proposal with coherent states for k=2 have been realized in experiments [18]. The proposal with N=1 could be tested, using standard set-ups involving photonic superposition states and polarising beam splitters. Even though not mesoscopic at N=1, it is remarked that the predictions are unchanged with *N*. By dividing Alice’s final transformation $U_{f}$ into two parts and comparing it with a mixed state as described in this paper, the wMR premise (2) can be tested. Similar mesoscopic interactions may be realizable for moderate *N* by applying the CNOT gates of the IBM computer [43].

## Data Availability

Data sharing is not applicable to this article.
